# *Toxoplasma gondii* in small exotic felids from zoos in Europe and the Middle East: serological prevalence and risk factors

**DOI:** 10.1186/s13071-019-3706-2

**Published:** 2019-09-11

**Authors:** Maike Lücht, Julia Stagegaard, Franz J. Conraths, Gereon Schares

**Affiliations:** 1 Friedrich-Loeffler-Institut, Institute of Epidemiology, Federal Research Institute for Animal Health, Südufer 10, 17493 Greifswald-Insel Riems, Germany; 2 Münchener Tierpark; Hellabrunn AG, Tierparkstr. 30, 81543 Munich, Germany; 3 Ree Park-Safari, Stubbe Søvej 15, 8400 Ebeltoft, Denmark

**Keywords:** *Toxoplasma gondii*, Serology, Felinae, Captive felids, Zoological gardens, Epidemiology, Risk analysis, Risk factors, Triatominae, *Dipetalogaster maxima*

## Abstract

**Background:**

*Toxoplasma gondii* infections and cases of clinical toxoplasmosis have been recorded in zoo animals. Wild felids in human care can serve as definitive hosts that shed oocysts, but also as intermediate hosts for the parasite. Some felid species, such as the Pallas’s cat (*Otocolobus manul*) or sand cat (*Felis margarita*), may suffer from clinically apparent toxoplasmosis. In the present study, our main aim was to assess risk factors for *T. gondii* infections in small exotic felids.

**Methods:**

A seroepidemiological study was conducted using the reduviid bug *Dipetalogaster maxima* for blood sample collection, a method previously evaluated on domestic cats. A total of 336 samples from 17 felid species were collected in 51 institutions, 48 of which were within Europe and the remaining three in the Middle East (United Arabic Emirates and Qatar). These samples were analyzed for *T. gondii* antibodies by immunoblotting and an immunofluorescent antibody test. Potential risk factors in zoos for seropositivity regarding *T. gondii* among members of the European Association of Zoos and Aquaria (EAZA) were evaluated using a questionnaire and individual data from the Zoological Information Management System (ZIMS).

**Results:**

The sampled felids showed an overall seroprevalence for *T. gondii* of 63%. The risk factor study including data of 311 small exotic cats of 10 species resulted in a final generalized linear mixed model comprised of five variables: the likelihood of seropositivity increased statistically significantly with “Age”, while feeding “Cattle: frozen” relative to “Cattle: fresh”, “Outdoor housing fenced in on all sides”, “Mesh size 2–5 cm” relative to “Mesh size > 5 cm” and “Wearing gloves: yes” had statistically significant protective effects.

**Conclusions:**

Wild felids, including endangered species, kept in human care in European and Middle Eastern institutions, are widely exposed to *T. gondii*. Risk factor analysis revealed that feeding previously frozen tissues, keeping animals in enclosures that are fenced on all sides using fences with small mesh sizes, and wearing gloves when working inside enclosures seem to be the most relevant protective measures to prevent *T. gondii* infections in these animals
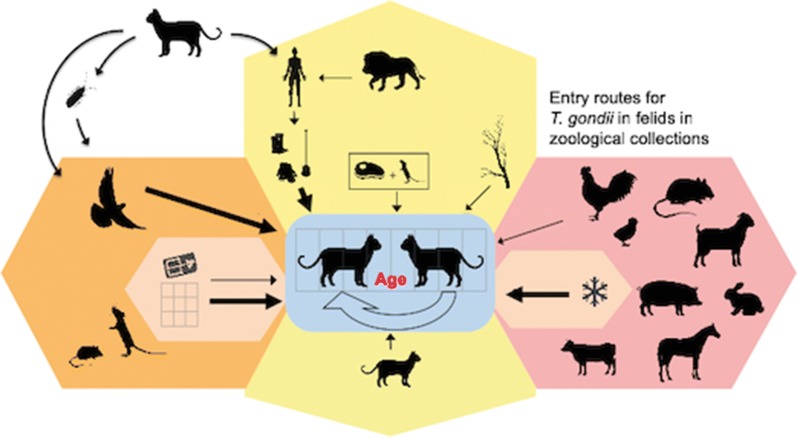
.

## Background

*Toxoplasma gondii* is a parasite, whose definitive hosts are felids [1]. Serological studies have shown that *T. gondii* infection is widely spread in wild animals [2–6]. Additionally, *T. gondii* infections are often reported in mammals and birds in human care in zoos [7–14].

At present, the infectivity of the different stages of *T. gondii* to exotic felids is unknown. A high susceptibility for *T. gondii* infection and subsequent toxoplasmosis is suspected for species that are rarely in contact with the parasite in the wild: Pallas’s cats (*Otocolobus manul*) in human care and, to a certain extent, sand cats (*Felis margarita*) have shown high mortality in cases where *T. gondii* was suspected to be the cause [15–18].

A mortality of 58% (14/24 kittens) was reported for newborn Pallas’s cat kittens born in human care in an Austrian zoo, where the suspected cause was an acute *T. gondii* infection [19]. In North America, the seroprevalence of *T. gondii* was 100% in nine Pallas’s cats in three zoos [15]. A mortality of 35% (6/17) due to acute toxoplasmosis was recorded in Pallas’s cat kittens in Denver Zoo. Since five kittens disappeared and were not available for necropsy, the mortality may have been as high as 65% (11/17) if those individuals that had died but could not be further investigated were also affected by toxoplasmosis [17]. In the Czech Republic, 12 fatal cases of suspected toxoplasmosis in Pallas’s cats were recorded between 2004 and 2013; in eight cases (66.6%), toxoplasmosis was confirmed [20]. The reasons for the increased susceptibility of Pallas’s cats for toxoplasmosis are not fully understood, but a study on Pallas’s cats conducted in Oklahoma, USA, suggested an immunodeficiency (congenital or acquired), since multiple diseases occurred in the examined population. In this study, an immunodeficiency similar to that caused by feline immunodeficiency virus (FIV) infection was suspected to play a role [21, 22]. It seems that the disease is usually asymptomatic in adults [23]; however, Dubey et al. [24] reported a case of fatal toxoplasmosis in an adult Pallas’s cat.

A serological study confirmed a high exposure of adult Pallas’s cats to *T. gondii* in North American zoos. More than 80% of the animals tested positive for antibodies to the parasite [18]. This contrasts with studies on wild Pallas’s cats in Mongolia and Russia, which suggested a low incidence of *T. gondii* infection in this species. In 2000–2001, 15 Pallas’s cats, 15 domestic cats and 45 prey animals were captured in Mongolia. Only two Pallas’s cats (13%) showed a positive *Toxoplasma*-antibody response in enzyme immunoassays (EIA) and no evidence for exposure to *T. gondii* was found in the domestic cats or prey animals [15]. In 2010 and 2011, 16 wild Pallas’s cats were caught in Russia close to the Mongolian border. As in the study of Brown et al. [15], only 13% of the individuals showed positive *T. gondii* antibody reactions using EIA [25].

It is suspected that animals like Pallas’s cats, which live in dry habitats with very severe winters and at high altitudes, rarely come into contact with *T. gondii* in nature. The climatic conditions in the natural habitat of Pallas’s cats may also reduce the viability of *T. gondii* oocysts. This seems to be the most obvious reason why this species has a minimal chance of being naturally exposed to *T. gondii* [15, 26]. In general, the prevalence seemed to be higher in areas with a warm climate and low altitude than in regions with a cold climate and high altitude. Furthermore, the prevalence was higher in areas with a climate of high humidity than in arid regions [27].

A similar situation can be observed for sand cats. The natural spread of *T. gondii* seems to occur less in hot and arid climates, the typical habitat of sand cats [27]. Lack of exposure to *T. gondii* during phylogeny might be one of the reasons for the increased susceptibility to infection or disease, possibly due to impaired immune reaction against *T. gondii* or lack of adaptation to the parasite. There are reports of deaths in sand cats that are suspected to have been caused by toxoplasmosis [16, 28].

The extent to which the European population of small exotic felids in human care are infected with *T. gondii* is presently unknown. Seroprevalences in captive felids were examined in Brazil, Thailand, the USA and the UAE. In Brazil, three independent studies were performed. In a first study, felids of eight species in Brazilian zoos were analyzed and an overall prevalence of 55% (472/865) was found [8]. A second study demonstrated a seroprevalence of 63.4% (102/161) in a sampled population of wild felids belonging to 14 different species [29]. A third study was performed at the Itaipu Binacional Wildlife Research Center. This study included felids of five species, among which a prevalence of 66.7% (38/57) was detected [30]. In Thailand, 12 feline species were tested and a total seroprevalence of 15.4% (21/136) was reported [31]. In the USA, samples of 17 species kept in human care were analyzed and a seroprevalence of 59% (35/59) was reported [32]. In the UAE, 29 Gordon’s wildcats (*Felis silvestris gordoni*) in human care were tested. All individuals were seropositive using the modified agglutination test (MAT) [33].

In domestic cats, the exposure risk can be reduced by keeping cats indoors to avoid ingestion of possibly infected rodents or birds and by implementing regular pest control to minimize the risk of contact with various potentially infected intermediate host species [27]. Meat should not be fed raw, but cooked until it has reached an internal temperature of 61 °C for at least 3.6 min [34] or stored frozen at − 12 °C for at least seven days to destroy tissue cysts [34–36]. In addition, litter boxes should be cleaned daily as oocysts need at least 24 hours to sporulate and become infective [1]. Dogs should be kept away from litter boxes to avoid the ingestion and passage of oocysts [37, 38].

Silva et al. [39] studied risk factors for seropositivity to *T. gondii* in wild Neotropical felids in human care from Brazil and concluded that the most effective way to reduce the risk of exposure would be to freeze meat to – 12 °C for more than one week before feeding it to the cats [39].

To evaluate the seroprevalence of *T. gondii* in European Association of Zoos and Aquaria (EAZA) zoos, a minimally invasive technique using reduviid bugs, which has been described by various authors, can be a valuable alternative to conventional blood sampling [40–46]. Reports of the use of reduviid bugs were found as early as in 1971, when they were tested as an alternative bleeding method for geckos instead of the common practice to cut the tip of tail off. Blood samples were used to investigate the protein profile of geckos [47, 48]. Later reduviid bugs were successfully used to collect blood for the determination of antibody titers for various disease such as rabies [44], rabbit hemorrhagic disease virus (RHDV) [45], tuberculosis (TB), bluetongue and brucellosis [49]. Reduviid bugs were furthermore used in endocrinologic studies [40, 46, 50, 51] and for genetic analysis [52]. Additionally, lymphocytes for karyological analysis were isolated from bug-derived blood to identify bat species [53].

One objective of this study was to estimate the prevalence of *T. gondii* in small exotic felids in EAZA zoos. A further objective was to determine putative risk factors for *T. gondii* seropositivity in these felids.

## Methods

### *Dipetalogaster maxima* used for blood sampling

**Fig. 1:**
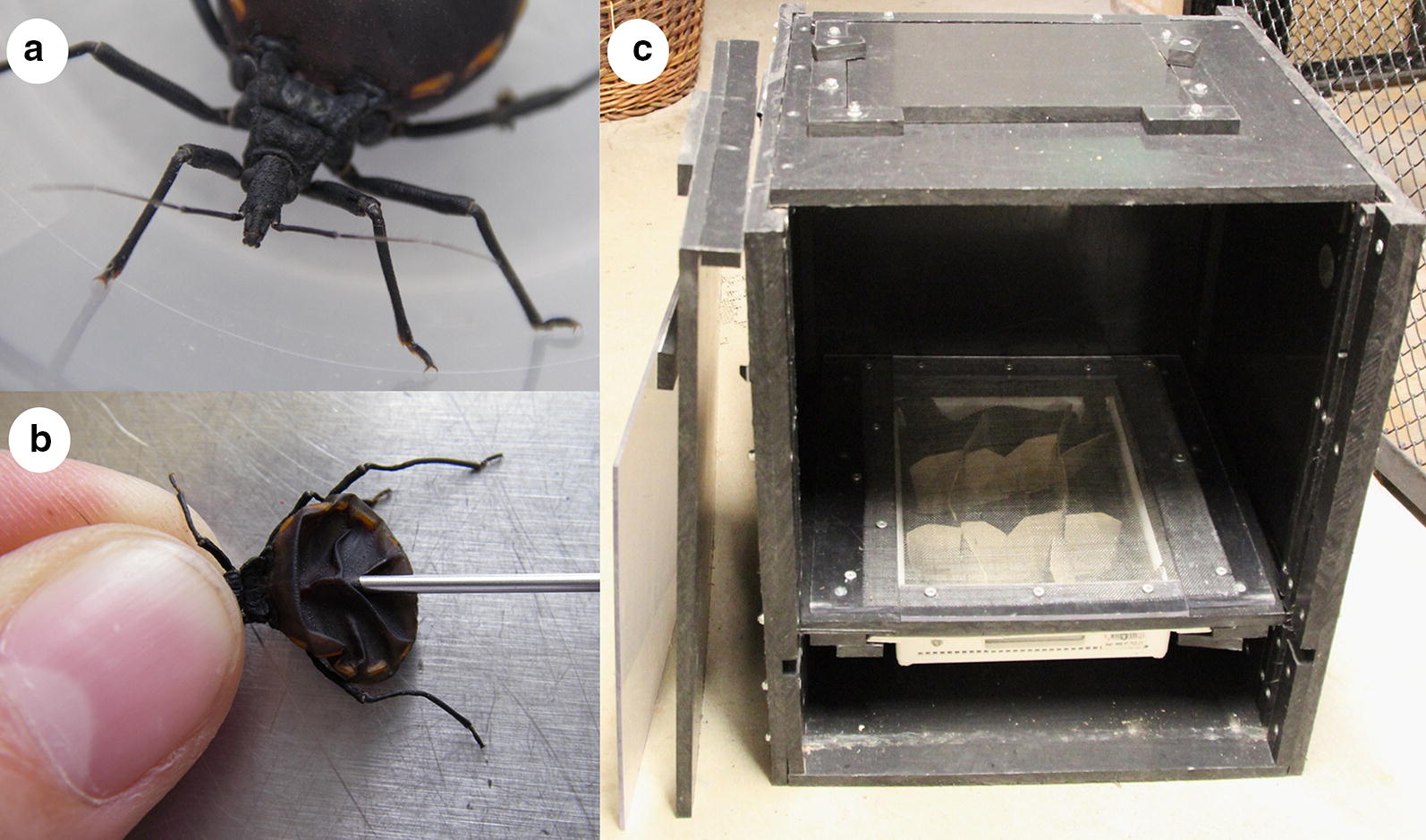
Blood sampling using reduviid bugs. **a** *Dipetalogaster maxima*, larval stage 5. **b** Blood aspiration from *D. maxima* using a 1.2 × 40 mm needle and a 3 ml syringe. Blood aspiration from the abdomen of the bug was performed from dorsal while holding the thorax of the bug. This approach facilitates the retrieval of the maximum amount of blood out of the bug. **c** Sample collection box used for *D. maxima*-based blood sampling. The box was built at the Ree-Park - Safari (Ebeltoft, Denmark). The drawer is equipped with a wire netting, stable enough to support the weight of a small felid and contains a little container (white in the picture). Two pieces of transversing cardboards were placed inside the bug-container allowing the bugs to climb upwards the host

The reduviid bug *Dipetalogaster maxima* (Fig. 1a) is the largest species of all triatomines [49] and thus convenient for use in blood sampling. *Dipetalogaster maxima* were purchased from Ruhr-Universität, Bochum, Germany, Faculty of Biology and Biotechnology, working group Zoology/Parasitology, where they were reared at a temperature of 26–28 °C, a relative humidity of 70% and with a photoperiod of 12/12 h (light/dark). During the time of rearing, they were kept in beakers (15 × 18 cm) covered by a nylon cloth. A commercial rubber ring was used to fix the nylon cloth to the beakers. To increase the area for the bugs to sit on, two pieces of transversing cardboards were provided. Filter paper was placed on the bottom of the beakers to soak the excretions of the bugs. One colony of 100 first-instar larvae was kept in a 2 l beaker until they reached adult stage. The bugs were fed on chicken blood for 1 h [49]. After feeding, *D. maxima* molts depending on the climatic conditions. At 26 ± 1 °C and 50–60% humidity, molting starts on day 14, 14, 16, 21 and 51 according to the larval stage, L1, L2, L3, L4 and L5, respectively [54]. Prior to blood sampling the bugs were fasted for at least eight weeks.

The development of *D. maxima* consists of five larval stages (L1–L5) until the imago hatches. The amount of blood ingested by the bug depends on the larval stage (up to 0.1 g, 0.2 g, 0.6 g, 1.2 g and 2.5 g from L1 to L5, respectively) [49], with the larvae increasing in mean total body length of about 0.8 cm (L1), 1.2 cm (L2), 1.5 cm (L3), 2.0 cm (L4) and 2.6 cm (L5) [55]. To obtain enough blood for analyses from each bug, large L4 or L5 larvae were used in this study, taking up approximately 0.8–1.2 ml of blood per bug. Each bug was used only once to rule out cross-contamination and transmission of diseases to other hosts. To assure that only completely “empty” bugs were used, individuals with a paper-thin abdomen were chosen. Waiting eight weeks after the last blood meal, i.e. until it was completely digested, avoided contamination of samples with bug hemolymph [56].

To keep the bugs at their preferred temperature of 26 °C and a humidity between 60% and 70%, they were housed and transported in an incubator (“Kunstglucke FB 50 E-Reptilien”, Jaeger, Wächtersbach, Germany) that was suitable for use inside a car. An adapter also made indoor use possible. Temperature and humidity were controlled using a digital thermo-hygrometer with two separate probes (Terra Exotica, Alfeld, Germany). Humidity was increased by placing a wet sponge inside the incubator if necessary.

### Validating the use of *D. maxima* for sampling blood for antibody detection

To examine the relationship between *T. gondii* antibody titers in plasma separated from blood collected *via* reduviid bugs and those measured in plasma obtained by conventional blood sampling, domestic cats were used. Blood samples from 70 domestic cats were collected after the owners’ consent had been obtained. Samples were taken only from cats that had been anaesthetized for other reasons at a small animal clinic. Anesthesia was necessary in these animals because of diagnostic procedures or treatments (e.g. castrations, wound or dental treatment) not related to this study. Anaesthesia was performed by intramuscular injection of 80 µg/kg medetomidin (Domitor^®^; Vetoquinol, Ismaning, Germany) and 7.5 mg/kg ketamin (Ketamin 10%^®^; bela-pharm GmbH & Co. KG, Vechta, Germany). All individuals were closely monitored during the procedure.

To obtain blood samples, *D. maxima* (Fig. 1a) were placed in a modified medical urine container. The lid of the container was closed with netting that allowed the proboscis of the bugs to cross the meshes and reach the cat. The container itself was divided into four chambers using two transversing cardboards, assembled to a cross. In each chamber, a single reduviid bug was placed. The readiness of the bugs to obtain a meal was assessed by blowing gently into the container. Only individuals that showed immediate interest by elevating their proboscis and searching for the source of potential prey were used to perform blood collection. The modified urine container was placed either on the lateral chest or the abdomen of the anesthetized cat depending on the procedure and the position of the patient, to ensure that the container did not disturb the medical procedure performed on the animal.

The time when each bug started obtaining blood was noted as well as the time when it detached fully engorged. Full engorgement took between 10 and 20 min. After that time, the blood was immediately obtained (Fig. 1b) from the first bug (sample Dm0). The other bugs were placed in a room at a temperature of about 23 °C. The blood sample from the second bug (sample Dm1) was obtained 1 h after engorgement had terminated. Immediately after the blood was withdrawn, it was transferred into a tube supplemented with lithium heparin (1.3 ml; Sarstedt, Nürnbrecht, Germany). The tubes were centrifuged in a Spectrafuge™ Mini centrifuge (Labnet International, Edison, NJ, USA) at 2000×*g* for 5 min and plasma was removed and kept frozen at − 20 °C until further analysis. After blood withdrawal, the bugs were killed by decapitation.

### Felids sampled in EAZA zoos

For the evaluation of the prevalence of *T. gondii* in EAZA zoos (including European and Middle Eastern zoos), only small cat species managed in a special breeding programme, like the EEP (European Endangered Species Programme) and the ESB (European Studbook) or at least monitored on a regular basis (yearly intervals) were chosen.

**Fig. 2:**
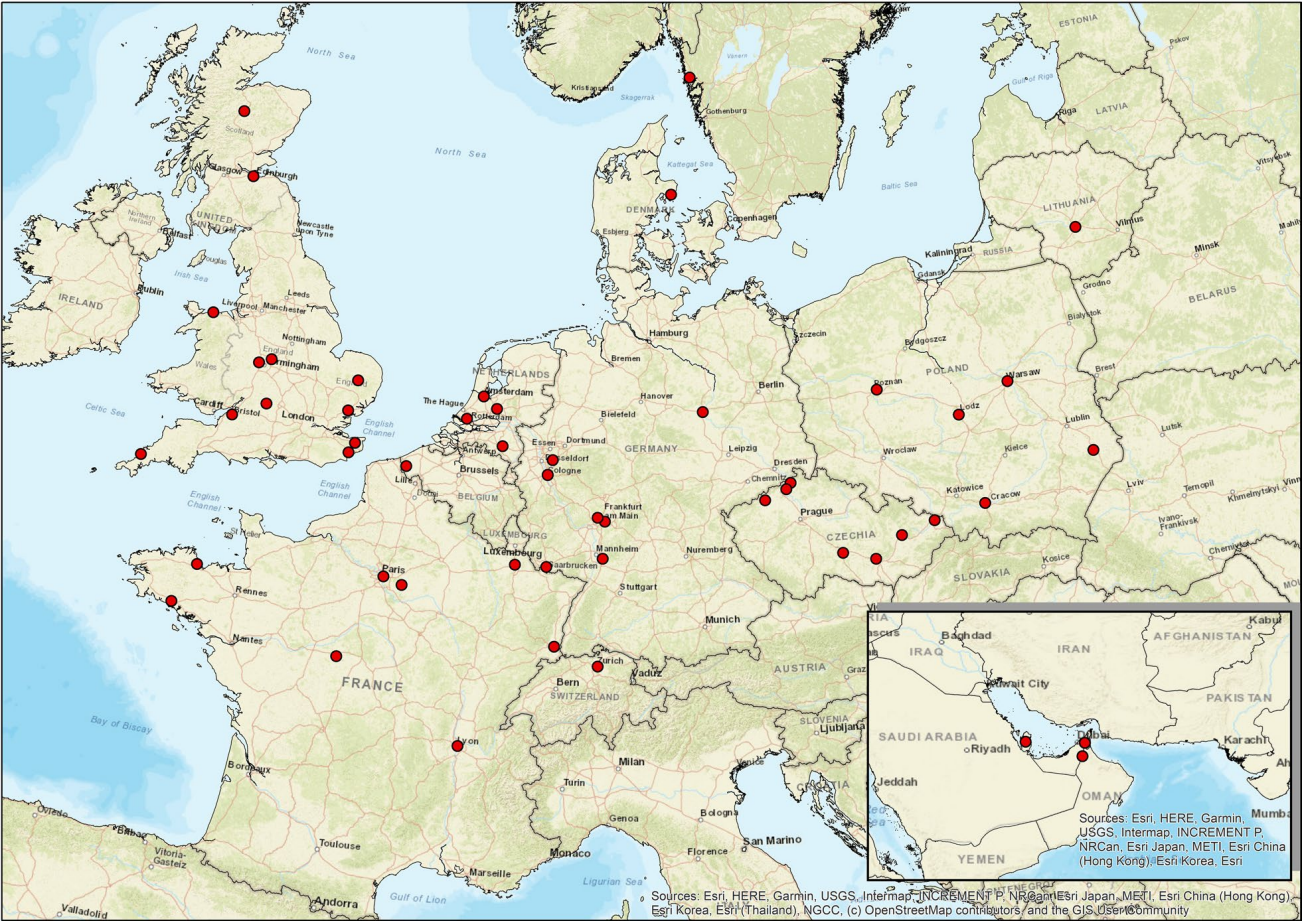
Geographical distribution of participating zoos

Ten species of small exotic cats were sampled in 51 EAZA zoos (Fig. 2): Asian golden cats (*Catopuma temminckii*), black-footed cats (*Felis nigripes*), fishing cats (*Prionailurus viverrinus*), Geoffroy’s cats (*Leopardus geoffroyi*), jaguarundis (*Puma yagouaroundi*), margays (*Leopardus wiedii*), oncillas (*Leopardus tigrinus*), Pallas’s cats (*Otocolobus manul*), rusty-spotted cats (*Prionailurus rubiginosus*) and sand cats (*Felis margarita*).

In addition to samples collected using reduviid bugs, samples provided by serum banks were analyzed. In these cases, samples from other species were also used, later on referred to as “others”. Additional samples came from the following species: one clouded leopard (*Neofelis nebulosa*), one Eurasian lynx (*Lynx lynx*), three Gordon’s wildcats (*Felis silvestris gordoni*), one jungle cat (*Felis chaus*), two ocelots (*Leopardus pardalis*), three servals (*Leptailurus serval*) and seven wildcats (*Felis silvestris*).

Bug-derived blood samples were obtained by placing two to four bugs inside the bug-container in the collection box (Fig. 1c). The drawer was then placed inside the box and the cat caught, or encouraged with food to move inside the box. The cats were left inside the box for no longer than 1 h to give them time to settle, so that the bugs could proceed with their blood meal. Blood was obtained immediately from the bugs and processed (Fig. 1b). If more than one bug successfully finished their meals, the plasma samples were pooled.

### Serological blood analysis

Antibodies specific for *T. gondii* were determined in an immunoblot based on the tachyzoite surface antigen TgSAG1 obtained by immunoaffinity chromatography using the mouse monoclonal antibody (mAb) IgG2a P30/3 (ISL, Paignton, UK) [57]. For Western blotting, purified TgSAG1 of *T. gondii* RH was used as described by Maksimov et al. [57] with few modifications. A quantity of 0.05 µg TgSAG1 was incubated in non-reducing sample buffer [2% (w/v) sodium dodecyl sulfate (SDS), 10 % (v/v) glycerol, 62 mM Tris-HCl, pH 6.8] for 1 min (94 °C), separated in 12% (w/v) SDS polyacrylamide minigels of 60 × 70 × 1 mm size and transferred to a polyvinylidene difluoride (PVDF) membrane (Immobilon-P, Merck Chemicals GmbH, Darmstadt, Germany) [58]. After transfer, the membrane was blocked using PBS-TG [PBS with 0.05 % (v/v) Tween 20 (Sigma-Aldrich, Deisenhofen, Germany) and 2% (v/v) liquid fish gelatine (Serva, Heidelberg, Germany)], cut into 50 strips and examined as described below. Cat plasma or serum was diluted 1:100 in PBS-TG. The reactivity of plasma samples with a single band of 30 kDa Mr was recorded (no reaction, very faint reaction, clear reaction). In all immunoblots, peroxidase conjugated anti-cat IgG (H + L) (Jackson Immunoresearch Laboratories, West Grove, PA, USA) was used diluted 1:250 in PBS-TG.

In addition, an immunofluorescent antibody test (IFAT) using the *T. gondii* strain RH was performed. Ten microliters of a suspension of cell culture-derived *T. gondii* RH strain tachyzoites (5 × 10^6^ ml^−1^) in phosphate-buffered saline (PBS) were used to sensitize IFAT slide wells. Slides were air-dried and stored frozen at − 20 °C until used. The slides were fixed with ice-cold acetone for 10 min and then incubated in PBS for 10 min. Cat plasma or serum was titrated in PBS in 2-fold steps starting at a plasma dilution of 1:50. The test was performed as described for *N. caninum* [59] with the following modification: anti-cat IgG (H&L) produced in goat and coupled to fluorescein-isothiocyanate (Rockland Immunochemicals, Pottstown, PA, USA) diluted 1:50 in PBS (including 0.2% Evans Blue as a counterstain) was used to detect primary antibodies. The slides were examined under an Olympus AHBT3 microscope (Olympus, Hamburg, Germany). Only complete peripheral fluorescence of the tachyzoite was considered specific. The positive cut-off was a titer of 1:100 [60].

A *Toxoplasma*-seropositive or -seronegative result was recorded when both tests (TgSAG1 recognized in the immunoblot and IFAT titer 1: ≥ 100) had the same result (both tests positive or negative). When the immunoblot showed an inconclusive result, the IFAT result was accepted as valid. In 25 cases, where the results of both tests differed, the final judgement was that the result was considered as not reliable. These individuals with an unclear serological result were excluded from further analysis.

### Data from the Zoological Information Management System and questionnaire

Individual data on each felid that took part in the study was gathered from the Zoological Information Management System (ZIMS). Data collected from ZIMS included the age of the animal or the date of birth, relevant identifiers (Studbook no., ZIMS ID, microchip no.), sex and life history (number of institutions the animal had lived in).

A standard questionnaire (Additional file 1: Figure S1**)** was designed to obtain further information about the zoos, the place of sampling and possible *T. gondii* infection routes for the felids. In addition to details about the animal collection (number of small exotic felids at the institution), information on animal keeping and husbandry was collected. Data on feeding and food storage, husbandry as in cleaning habits and enclosure interior, details about pest control, and information about known incidences of toxoplasmosis as well as other typically cat-associated disease (FHV1, Calicivirus, FeLV, FIP, FIV, feline distemper) were also recorded. Furthermore, the questionnaire contained questions about prophylactic measures such as vaccinations and parasite control.

To make the results comparable, questions were designed closed-ended if possible. Multiple answers were accepted. Questionnaires were filled in by a representative of the institution or by the author during the visit of the institution.

### Statistical tests and software used for analysis

The statistical analysis aimed at the comparison of paired data obtained for domestic cats on venous plasma (V) and bug-derived plasma (Dm0, Dm1), whereby each individual time point was analyzed separately. Statistical tests were performed in the IBM Statistical Package for the Social Sciences (SPSS) version 24 (SPSS Inc., Chicago, IL, USA). In a first step, data were analyzed for a normal distribution using the Shapiro-Wilk test. As the data were not normally distributed, the level of correlation was determined by the Spearmanʼs rank correlation test. The level of correlation was only assessed if *P* < 0.05.

In the statistical risk factor analysis, the serological results for *T. gondii* of wild felids in human care in EAZA zoos were considered as the dependent variable. Since felids came from different zoos, random effects that might have been caused by different zoos were included in the models.

**Fig. 3:**
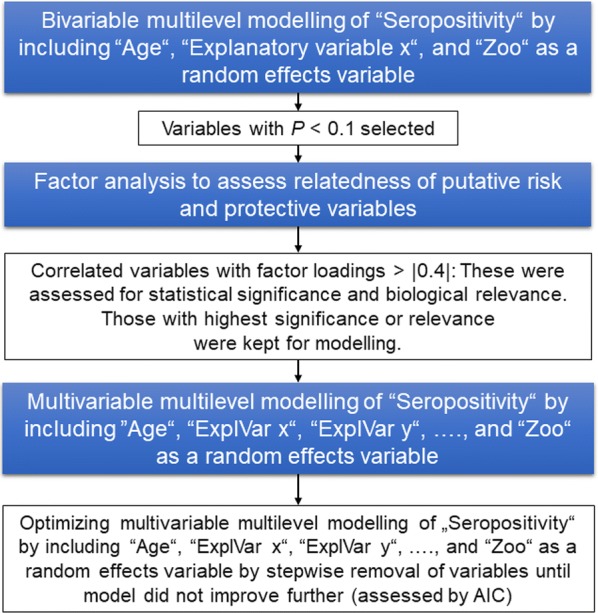
Flow chart on data analysis to assess potential risk factors for *Toxoplasma*-seropositivity

For the identification of potential risk factors, bivariable-multilevel-modeling [generalized linear mixed modeling fit by maximum likelihood (Laplace approximation)] was performed using R (http://www.R-project.org) version 3.3.1, by applying the package *lme4* (Fig. 3). As seropositivity clearly increased with age and had to be regarded as an important effect-modifying explanatory variable, data on age (in years) of individual animals were included into each of the bivariable models calculated. Animals, for which no sampling date was available (*n* = 13) were excluded from the analysis.

To find out whether the input variables were independent of each other in the dataset, a factor analysis (assuming a maximum number of possible factors, respectively) was performed using the command “factanal” (scores = ‛Bartlettʼ). Absolute factor loadings of > 0.4 were regarded as an indication of dependence between explanatory variables (ExplVar) (Fig. 3). Dependent variables were reduced to one per model by choosing the variable with the best (i.e. lowest) Akaike information criterion (AIC) in the bivariable analysis and in two cases by excluding those variables that were regarded as less relevant to have a biological effect on *T. gondii* seropositivity. This was the case for a putative dependence between “MeshSize” and “Rabies vaccination” suggested purely by statistical analysis, as the biological relevance of a rabies vaccination for seropositivity to *T. gondii* remained unclear. For the putative dependencies between feeding “Cattle” tissues and “Litters within 1 year” and feeding “Mice” and “Deworming interval”, the assumption was made that a feeding-related variable has a higher biological plausibility than the breeding-related variable “Litters within 1 year” or the general health related variable “Deworming interval”.

In a last step, all relevant and independent variables were included into a generalized linear mixed model (multivariable-multilevel-model) to determine potential risk factors for *Toxoplasma*-seropositivity in wild felids in human care (Fig. 3). After optimization by a stepwise elimination of those variables that, if removed, did not cause an increase in AIC, the final linear mixed model was generated (Fig. 3).

## Results

### Validation of the *Toxoplasma gondii* serology performed with plasma samples obtained by using reduviid bugs

**Fig. 4:**
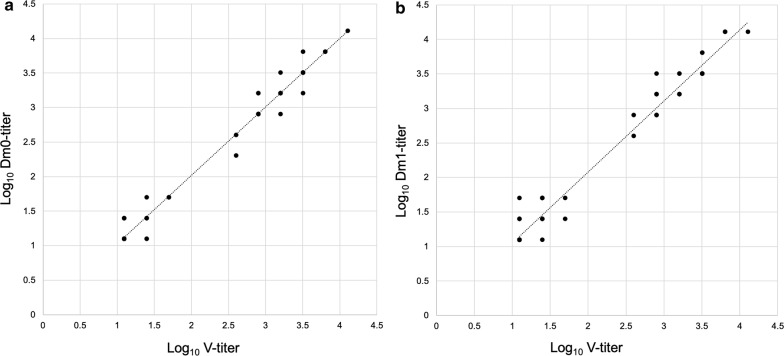
Correlation of *Toxoplasma gondii* titer values in venous (V) and bug-derived plasma, in Dm0, immediate after sampling (**a**) and in Dm1, sampling one hour after the engorgement was completed (**b**). Spearmanʼs test, V *vs* Dm0: *r* = 0.952, *P* < 0.001; V *vs* Dm1: *r* = 0.954, *P* < 0.001; linear regression lines were drawn using Microsoft Excel 2010

Conventionally sampled plasma (V) or plasma sampled with reduviid bugs (Dm0, Dm1) from 70 domestic cats was tested for *T. gondii*-specific antibodies by both immunoblot and immunofluorescent antibody tests (Additional file 2: Table S1). All individuals that showed a positive titer in the venous blood were also positive in all samples taken by reduviid bugs. Similarly, all animals with *T. gondii-*negative V-samples tested also negative in the samples obtained through reduviid bugs. The data on IFAT titers failed to show a normal distribution and were therefore statistically analyzed using the Spearmanʼs rank correlation. The titers determined in venous plasma and the titers determined in bug-derived samples were statistically significantly correlated (*r* ranging between 0.952–0.954, *P* ≤ 0.001) (Fig. 4).

### Outcome of previous *T. gondii* diagnostic tests in EAZA zoos

The zoos were asked if they had performed any tests for *T. gondii* over the last five years. In total, 291 animals of various species had been tested, 69.8% with a *T. gondii-*seropositive and 30.0% with a negative result. In zoos that reported details, 28 of the seropositive animals were felids, 15 were monkeys, 5 were marsupials and 35 belonged to various other species (Table 1).

**Table 1:** Numbers and proportions of animals that had tested positive for antibodies to *Toxoplasma gondii* over the last five years in zoos

	Zoos that performed *T. gondii* tests	Total no. of animals tested	Felids	Marsupials	Monkeys	Other
Total number	25^a^	129	62	11	17	39
No. of animals with a positive test result	15	83	28	5	15	35
Proportion (%)	60.0	64.3	45.2	45.5	88.2	89.7

^a^An additional zoo reported the examination of 162 animals for *T. gondii*. Of these, 77 felids and 43 non-felid species tested positive. Because the report of this zoo lacked details, data could not be included in the table

### Serological results in wild, small felids in human care in EAZA zoos

In total, 336 samples from 17 felid species, collected in 51 institutions were analyzed for *T. gondii* antibodies by immunoblot and IFAT. The tests revealed 196 positives and 115 negatives; 25 serological results were excluded from further analysis due to differences in the results of immunoblot and IFAT. From 311 animals with an unambiguous serological result, 63.0% showed a positive and 37.0% a negative antibody response (Additional file 3: Table S2).

### Statistical association between *T. gondii* seropositivity and individual animal data on species, sex and age

Individual animal characteristics were represented by data collected from ZIMS including date of birth, sex and life history (animal-origin) (Additional file 3: Table S2).

The highest percentage of *T. gondii*-seropositive animals was found in Pallas’s cats with 90.4% and in rusty-spotted cats with 96.4%. Both values were much higher than the mean proportion of *T. gondii* seropositive results in all felids tested (63%). In contrary to this, black-footed cats showed a considerably lower percentage of seropositive animals than the remaining species (26.7%) (Table 2).

**Table 2:** Serological results for *Toxoplasma gondii* in wild felids in human care stratified by species

Species	Total no. of samples	No. of positive samples (%)
Asian golden cat (*n* = 2)	2	1 (50.0)
Black-footed cat (*n* = 3)	15	4 (26.7)
Fishing cat (*n* = 15)	40	22 (55.0)
Geoffroy’s cat (*n* = 14)	33	16 (48.5)
Jaguarondi (*n* = 5)	9	5 (55.6)
Margay (*n* = 7)	19	9 (47.4)
Oncilla (*n* = 3)	9	6 (66.7)
Pallas’ cat (*n* = 22)	52	47 (90.4)
Rusty-spotted cat (*n* = 3)	28	27 (96.4)
Sand cat (*n* = 15)	87	47 (54.0)
Other (*n* = 5)	17	12 (70.6)
Total (*n* = 50)	311	196 (63.0)

*Abbreviation*; n, number of zoos

**Fig. 5:**
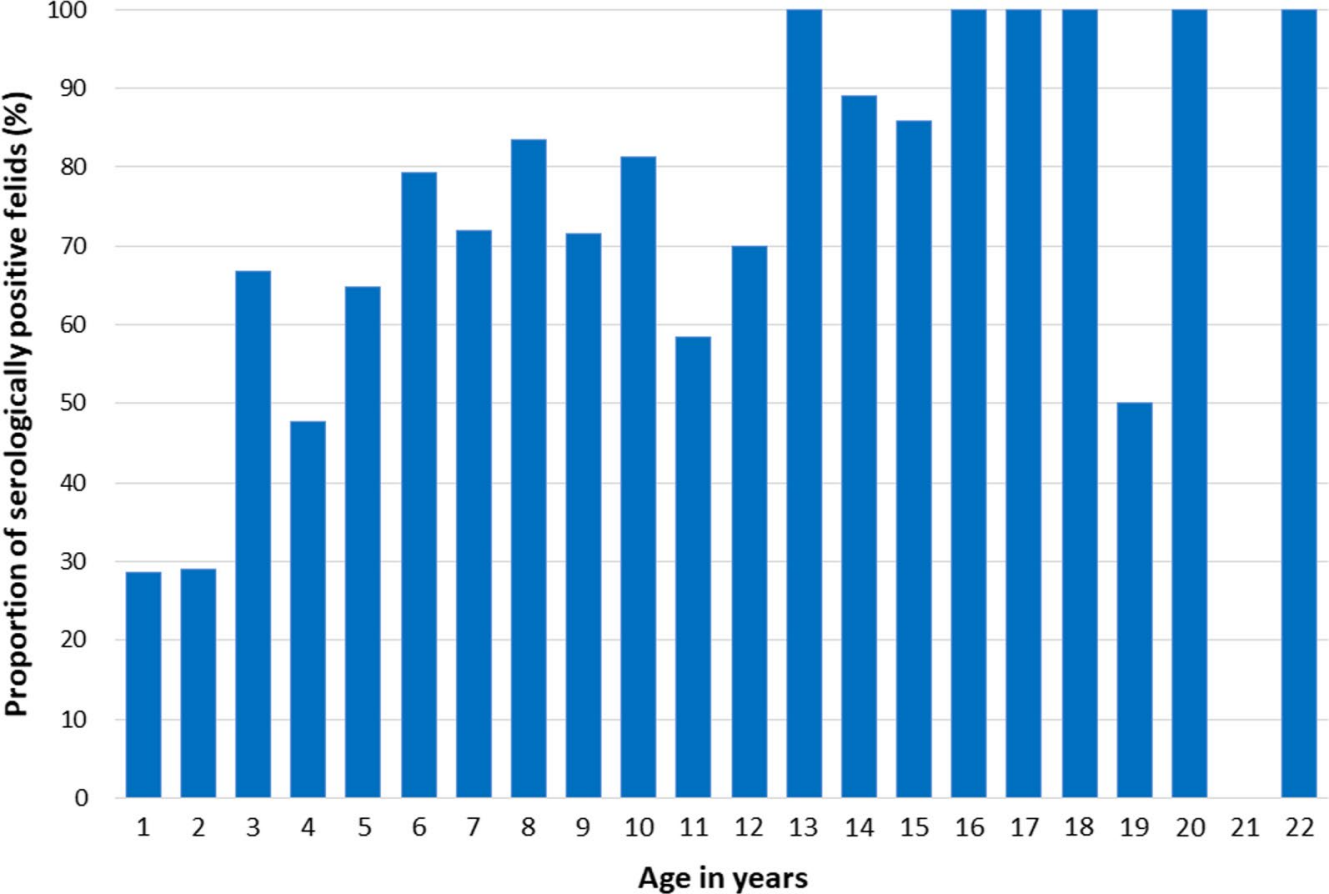
Proportions of *Toxoplasma gondii*-serologically positive wild felids in human care stratified by age

When the serological results were stratified by the age of the animals, the proportions of seropositive cats increased with the age (Fig. 5). Furthermore, a generalized linear mixed model fit by maximum likelihood (Laplace approximation) including random effects for the different zoos revealed a statistically significant effect of age (*P* < 0.001, Table 3, Model 1). Based on this finding, we concluded that data were biased by the age of the felids. Thus, univariable statistics was avoided because it became obvious that the age of the animals represented the major factor related to the probability of the animals to be seropositive for *T. gondii*. The data were thus exclusively analyzed by multilevel modeling [generalized linear mixed model fit by maximum likelihood (Laplace approximation)], with “Age” (in years) as an effect-modifying variable and the zoo the animal lived in as a random effects variable (Fig. 3, Tables 3, 4). A first generalized linear mixed model (including age and zoo) revealed that male animals had an increased risk of testing seropositive (Table 3).

**Table 3:** Fixed effects in generalized linear mixed models to determine potential risk factors for *Toxoplasma gondii-*seropositivity in wild felids in human care. Data were analyzed by bivariable generalized linear mixed modelling including “Age” (years) as effect modifier and “Zoo” as random effects variable in modelling *T. gondii*-seropositivity. The Akaike information criterion (AIC) was used to characterize the relative model quality

Category	Model (AIC, model fit)	Variable	Odds ratio (95% CI)	*z-*value	*P*-value
Individual risk factors	1 (357.4)	(Intercept)	0.523 (0.33–0.83)	− 2.718	0.0066**
		Age	1.219 (1.21–1.23)	80.86	< 0.001***
	2 (354.8)	(Intercept)	0.401 (0.19–0.86)	− 2.363	0.0181*
		Age	1.218 (1.12–1.32)	4.640	< 0.001***
		Sex: female (ref.)			
		Sex: male	1.779 (1.01–3.15)	1.976	0.0482*
Food-related risk factors	3 (300.6)	(Intercept)	1.309 (0.46–3.76)	0.500	0.6171
		Age	1.224 (1.12–1.34)	4.278	< 0.001***
		Mice: fresh (ref.)			
		Mice: fresh/frozen	0.240 (0.06–0.99)	− 1.975	0.0482*
		Mice: frozen	0.308 (0.08–1.13)	− 1.776	0.0757
		Mice: no	0.407 (0.08–2.00)	− 1.109	0.2675
	4 (301.4)	(Intercept)	1.474 (0.40–5.44)	0.582	0.5609
		Age	1.231 (1.12–1.35)	4.325	< 0.001***
		Rodents: fresh (ref.)			
		Rodents: fresh/frozen	0.277 (0.06–1.32)	− 1.614	0.1066
		Rodents: frozen	0.250 (0.05–1.19)	− 1.746	0.0809
		Rodents: no	0.445 (0.05–4.22)	− 0.705	0.4807
	5 (296.6)	(Intercept)	0.986 (0.25–3.97)	− 0.019	0.9845
		Age	1.232 (1.12–1.35)	4.327	< 0.001***
		Ruminants: fresh (ref.)			
		Ruminants: fresh/frozen	0.792 (0.14–4.60)	− 0.260	0.7947
		Ruminants: frozen	0.158 (0.03–0.85)	− 2.146	0.0319*
		Ruminants: no	0.763 (0.17–3.43)	− 0.353	0.7242
	6 (296.4)	(Intercept)	1.157 (0.26–5.10)	0.192	0.8478
		Age	1.232 (1.12–1.35)	4.331	< 0.001***
		Cattle: fresh (ref.)			
		Cattle: fresh/frozen	0.675 (0.11–4.23)	− 0.420	0.6744
		Cattle: frozen	0.135 (0.02–0.80)	− 2.210	0.0271*
		Cattle: no	0.629 (0.13–3.07)	− 0.573	0.5667
	7 (301.9)	(Intercept)	1.725 (0.36–8.17)	0.686	0.4925
		Age	1.222 (1.11–1.34)	4.195	< 0.001***
		Fowl: fresh (ref.)			
		Fowl: fresh/frozen	0.354 (0.07–1.92)	− 1.204	0.2285
		Fowl: frozen	0.221 (0.04–1.13)	− 1.817	0.0693
		Fowl: no	0.371 (0.02–5.62)	− 0.715	0.4746
Breeding and housing-related risk factors	8 (321.4)	(Intercept)	0.188 (0.06–0.59)	− 2.859	0.0043**
		Age	1.229 (1.12–1.34)	4.526	< 0.001***
		Litters within 1 year-Null (ref.)			
		Few litters (1–2) within 1 year	3.767 (1.12–12.7)	2.365	0.0180*
		Many litters (≥ 3) within 1 year	5.097 (1.32–19.7)	2.138	0.0325*
	9 (324.4)	(Intercept)	0.224 (0.06–0.81)	− 2.279	0.0227*
		Age	1.222 (1.12–1.34)	4.392	< 0.001***
		Litters within 5 years: null (ref.)			
		Few litters within 5 years, 1–9	2.311 (0.62–8.67)	2.115	0.0345*
		Many litters within 5 years, ≥ 10	4.191 (1.11–15.8)	1.242	0.2143
	10 (297.9)	(Intercept)	0.662 (0.30–1.45)	− 1.031	0.3026
		Age	1.236 (1.12–1.36)	4.364	< 0.001***
		New World monkeys close by: no (ref.)			
		New World monkeys close by: yes	0.309 (0.08–1.16)	− 1.738	0.0822
	11 (298.4)	(Intercept)	1.568 (0.41–6.06)	0.653	0.5140
		Age	1.216 (1.11–1.33)	4.196	< 0.001***
		Outdoor housing fenced in on all sides: no (ref.)			
		Outdoor housing fenced in on all sides: yes	0.297 (0.08–1.15)	− 1.759	0.0785
	12 (291.5)	(Intercept)	2.169 (0.61–7.68)	1.177	0.2393
		Age	1.208 (1.10–1.32)	4.247	< 0.001***
		Mesh size > 5 cm (ref.)			
		Mesh size < 2 cm	0.511 (0.10–2.71)	− 0.969	0.3326
		Mesh size 2–5 cm	0.204 (0.05–0.79)	− 2.416	0.0157*
Hygiene related risk factors	13 (295.1)	(Intercept)	0.862 (0.40–1.85)	− 0.379	0.7044
		Age	1.225 (1.12–1.34)	4.347	< 0.001***
		Wearing gloves: no (ref.)			
		Wearing gloves: yes	0.286 (0.10–0.79)	− 2.429	0.0151*
General health related risk factors	14 (248.5)	(Intercept)	0.204 (0.07–0.61)	− 2.829	0.0047**
		Age	1.256 (1.13–1.40)	4.196	< 0.001***
		Rabies vaccination: no (ref.)			
		Rabies vaccination: yes	4.910 (1.35–17.8)	2.417	0.01563*
	15 (175.8)	(Intercept)	0.048 (0.00–0.68)	− 2.243	0.024903*
		Age	1.307 (1.12–1.52)	3.417	0.000633***
		Deworming interval (months)	1.449 (0.98–2.14)	1.876	0.060723

*Abbreviation*: ref., reference

* *P* ≤ 0.05, ** *P* ≤ 0.01, *** *P* ≤ 0.001

**Table 4:** Summary of a factor analysis to assess the dependency of variables that revealed statistical significance in the bivariable generalized linear mixed modelling including “Age” (years) as effect modifier and “Zoo” as random effects variable in modeling *Toxoplasma gondii*-seropositivity in wild felids in human care (Table 3, detailed information in Additional file 7: Tables S6–S8)

Factor analysis models (phase of analysis)	Factor	Not excluded from further analysis	Excluded from further analysis (reason for exclusion)
Model 1 (initial)	1	Feeding: mice	Feeding: rodents, Feeding: fowl (lower statistical significance in bivariable risk factor analysis than Feeding: mice)
	2	Feeding: cattle	Feeding: ruminants (lower statistical significance in bivariable risk factor analysis than Feeding: cattle)
	3	Breeding: litters within 1 year	Breeding: litters within 5 years (lower statistical significance in bivariable risk factor analysis than Breeding: litters within 1 year)
	4	Housing: mesh size	General health: rabies vaccination (lower biological relevance than Housing: mesh size)
Model 2 (subsequent)	1	Feeding: cattle	Breeding: litters within 1 year (lower biological relevance than Feeding: cattle)
Model 3 (subsequent)	3	Feeding: mice	Deworming interval: month (lower biological relevance than Feeding: mice)

*Notes*: Variables with absolute loadings > 0.4 in factor analysis (Additional file 7: Tables S6-S8) were regarded as dependent. The initial model (Model 1) included all statistically significant variables in the bivariable analysis. The subsequent models (Models 2, 3) included only variables that were not excluded on the basis of the results obtained in the initial factor analysis model

### Identification of potential risk factors

**Fig. 6:**
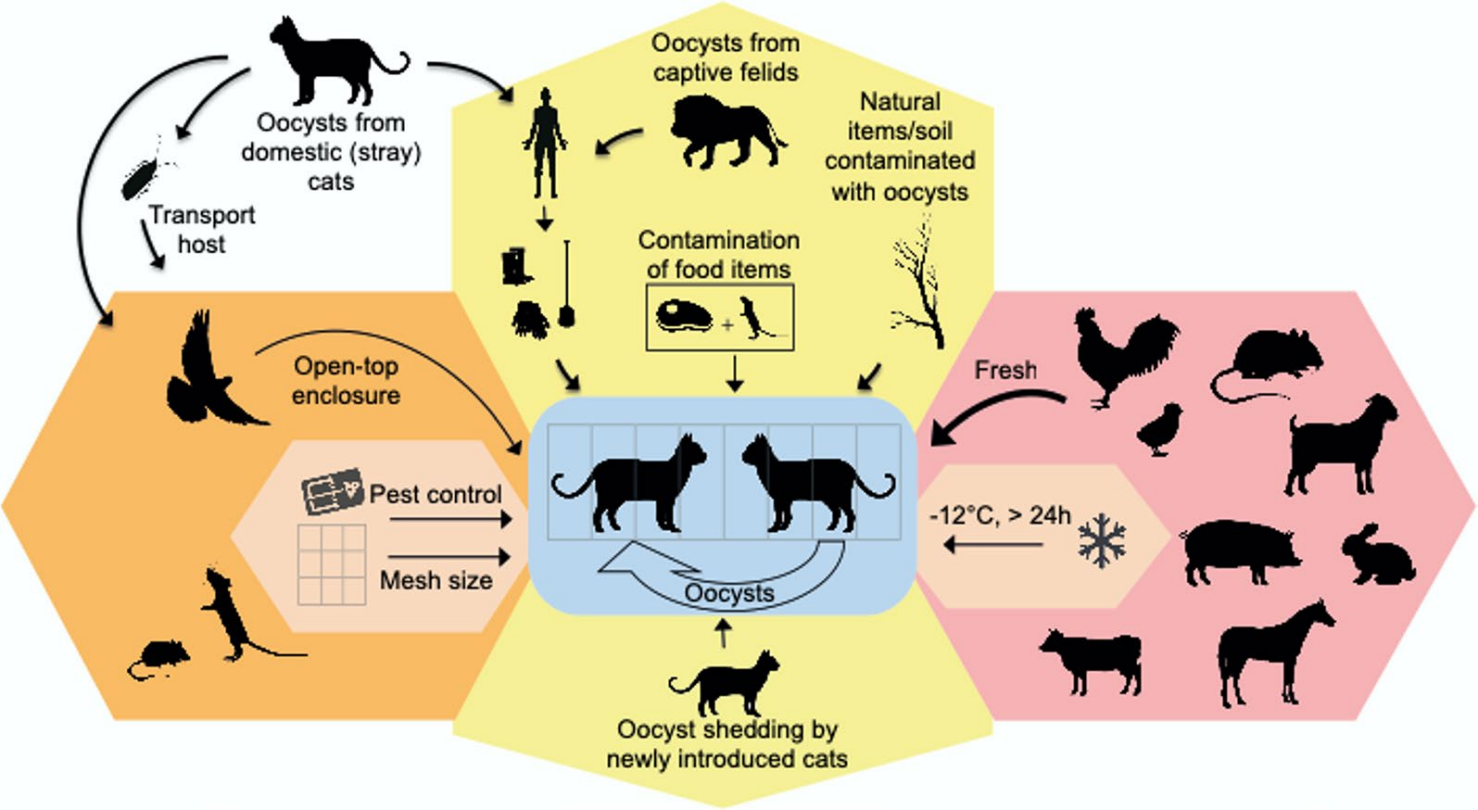
Potential entry routes for *Toxoplasma gondii* in zoos and institutions keeping felids in human care

Various routes may contribute to the infection of wild felids in human care with *T. gondii*. Different entry routes such as food, predation, hygiene and animal transport are displayed in Fig. 6.

In addition to characteristics of the individual animals (Additional file 3: Table S2), details on zoo-specific conditions (Additional file 4: Table S3) were collected using a structured questionnaire. Questionnaire data were divided into food-related (food and food preparation/storage), breeding and housing-related, hygiene- and general health-related variables and used for statistical analysis (Additional file 5: Table S4). A summary on serological findings stratified for individual animal and zoo-specific conditions is provided as Additional file 6: Table S5.

In a first step, a generalized linear mixed model was calculated that included seropositivity as the dependent variable and, in addition to age, all factors previously mentioned. In all models, age was retained as a statistically significant explanatory variable (Table 3).

In addition to age, 15 further variables including sex, variables characterizing feeding or treatment of food (e.g. feeding mouse tissues, cattle tissues or tissues from fowl), keeping other zoo animals, e.g. New World monkeys (NWM) close to felids, hygiene (e.g. wearing gloves), housing (e.g. outdoor housing, mesh-size of enclosures), health measures [e.g. rabies vaccination, deworming intervals (in months) and the numbers of litters produced by felids in the zoo (during the last year or during the past five years] had a statistically significant effect (*P* < 0.1) on the seropositivity of wild felids in human care according to generalized linear mixed models (Table 3).

Feeding mice, rodents, tissues from cattle, ruminants or fowl in a fresh condition was always the reference to analyze the risk of individual felids testing seropositive for *T. gondii*. Relative to these references, feeding these tissues after freezing had a statistically significant protective effect (*P* < 0.05 for meat from cattle and ruminants and *P* < 0.1 for carcasses of mice, rodents and fowl) (Table 3). Relative to the reference, mouse carcasses fed either fresh or previously frozen had a statistically significant protective model effect as well (*P* < 0.05) (Table 3).

Among the breeding and housing-related variables (Table 3), no litters born either within one or within five years prior to sampling were the references for analyzing the risk of individual felids testing seropositive for *T. gondii*. Relative to these references, “Few litters” born (1–2 within one year, or 1–9 within five years) proved to be statistically significant risk factors (*P* < 0.05). The same effect was found when three or more litters were born during the last year (*P* < 0.05).

Keeping “NWM close by” or keeping animals in “Outdoor enclosures fenced in on all sides” showed statistically significant protective effects (*P* < 0.1) compared to the references (“NWM close by: no”, “Outdoor housing fenced in on all sides: no”). A mesh size of 5 cm and more (characterizing fencing) was the reference to analyze the risk of individual felids testing seropositive for *T. gondii*; “Mesh size 2–5 cm” showed a statistically significant protective effect (*P* < 0.05).

Applying general health and hygienic measures such as wearing gloves or rabies vaccination had statistically significant protective effects on the risk of individual felids testing seropositive for *T. gondii* as compared to the references (“Wearing gloves: no”, “Rabies vaccination: no”; *P* < 0.05) (Table 3).

To find out whether the input variables were independent of each other in the dataset, a factor analysis was done for all variables listed in Table 4 (details of the factor analysis are provided in Additional file 7: Tables S6–S8), i.e. all variables that were statistically significant in bivariable generalized linear mixed models including “Age” as an effect modifier and “Zoo” as a random effects variable in modeling *T. gondii*-seropositivity in wild felids in human care (Table 4).

Finally, the model was optimized by a stepwise elimination of those variables that, if removed, did not cause an increase in AIC. The full model (including “Age”, “Sex”, “Mice”, “Cattle”, “NWM close by”, “Wearing gloves”, “Outdoor housing fenced in on all sides”, “Mesh size”, “Deworming interval”) had an AIC of 283.2. The final linear mixed model had an AIC of 276.8 and comprised the five variables “Age”, feeding “Cattle” tissues, “Outdoor housing fenced in on all sides”, “Mesh size” of enclosures and “Wearing gloves” (Table 5). In this model, the probability of seropositivity increased statistically significantly with the age of the animals. Feeding “Cattle: Frozen” relative to “Cattle: Fresh”, “Outdoor housing fenced in on all sides: yes”, “Mesh size 2–5 cm” relative to “Mesh size > 5 cm” and “Wearing gloves: yes” had a protective effect.

**Table 5:** Fixed effects in the final optimized generalized linear mixed models to determine potential risk factors for *T. gondii-*seropositivity in wild felids in human care. Modeling was performed assuming random effects by the variable “Zoo”

Model (AIC, model fit)	Category	Variable	Odds ratio (95% confidence interval)	z value	*P*-value
15 (276.8)		(Intercept)	16.82 (2.36–120)	2.818	0.005**
	Individual	Age	1.232 (1.13–1.34)	4.725	< 0.001***
	Food-related	Cattle: fresh (ref.)			
		Cattle: fresh/frozen	0.507 (0.14–1.85)	− 1.028	0.304
		Cattle: frozen	0.143 (0.04–0.57)	− 2.756	0.006**
		Cattle: no	0.397 (0.12–1.37)	− 1.462	0.144
	Housing	Outdoor housing fenced in on all sides: no (ref.)			
		Outdoor housing fenced in on all sides: yes	0.261 (0.08–0.88)	− 2.166	0.030*
	Housing	Mesh size more > 5 cm (ref.)			
		Mesh size < 2 cm	0.547 (0.14–2.20)	− 0.849	0.396
		Mesh size 2–5 cm	0.317 (0.10–0.99)	− 1.982	0.047*
	Hygiene	Wearing gloves: no (ref.)			
		Wearing gloves: yes	0.419 (0.20–0.90)	− 2.239	0.025*

*Notes*: Optimization of modeling was started with a full model [including all variables with a statistically significant effect (*P* < 0.1) in an initial bivariable generalized linear mixed model always including “Age” (years) in addition to the variable in question and proven independent by factor analysis] (Table 4). The Akaike information criterion (AIC) was used to characterize relative model quality. The full model had an AIC of 283.2. Optimization of the full model was done by a stepwise elimination of those variables that, if removed, did not cause an increase of AIC

*Abbreviations*: ref., reference

* *P* ≤ 0.05, ** *P* ≤ 0.01, *** *P* < 0.001

## Discussion

### Seroprevalence of antibodies to *T. gondii* in small exotic felids in European zoos

With 63.0% *T. gondii*-seropositive results in zoo felids, the seroprevalence for *T. gondii* was remarkably high in the tested felids. Worldwide, the seroprevalence for *T. gondii* in domestic cats (*Felis catus*) has been estimated as 30–40% [1]. In other studies, seroprevalences ranged from 15.4% to 59% among wild felids in human care (Table 6). The varying proportions between different species were interesting. While Pallas’s cats (90.4%) and rusty-spotted cats (96.4%) showed high proportions of seropositivity, the proportions of *T. gondii-*seropositive animals were considerably lower in other species, e.g. the black-footed cat (26.7%).

**Table 6:** Prevalence of *Toxoplasma gondii* in small cat species sampled in the present study compared with data from other studies

Species	Test	No. of samples	Proportion of positive samples (%)	Location	Reference
Geoffroy’s cat (*Leopardus geoffroyi*)	IB + IFAT	33	48.5	Europe	Present study
	Molecular/DNA	22	27.3	Brazil	[97]
	ELISA	8	25.0	Bolivian Chaco	[98]
	MAT	12	83.3	Brazil	[39]
	IFAT	1	0.00	California, USA	[32]
	MAT	1	100.0	Brazil	[30]
Jaguarundi (*Puma yagouaroundi*)	IB + IFAT	9	55.6	Europe	Present study
	MAT	2	50.0	Mexico	[13]
	IFAT	25	40.0	Brazil	[29]
	Molecular/DNA	22	40.9	Brazil	[97]
	ELISA	1	100.0	USA	[99]
	IFAT	1	100.0	Brazil	[100]
	IFAT	1	100.0	Czech Republic/Slovak Republic	[7]
	MAT	99	46.5	Brazil	[39]
	IHA + MAT	2	50.0	Brazil	[101]
	IFAT	2	0.00	California, USA	[32]
	MAT	3	66.7	Brazil	[30]
Margay (*Leopardus wiedii*)	IB + IFAT	19	47.4	Europe	Present study
	IFAT	4	100.0	Brazil	[29]
	Molecular/DNA	10	60.0	Brazil	[97]
	MAT	2	50.0	Guatemala	[102]
	IHA	2	0.00	California, USA	[10]
	MAT	63	54.0	Brazil	[39]
	IHA + MAT	1	100.0	Brazil	[101]
	MAT	17	58.8	Brazil	[30]
Oncilla (*Leopardus tigrinus*)	IB + IFAT	9	66.7	Europe	Present study
	MAT	2	0.00	Mexico	[13]
	IFAT	35	62.9	Brazil	[29]
	Molecular/DNA	28	28.6	Brazil	[97]
	IFAT	1	100.0	Bolivia	[103]
	MAT	131	50.4	Brazil	[39]
	DT	9	66.7	Brazil	[104]
	MAT	22	68.2	Brazil	[30]
Asian golden cat (*Catopuma temminckii*)	IB + IFAT	2	50.0	Europe	Present study
	MAT	2	50.0	Australia	[105]
	IHA	3	33.3	California, USA	[10]
	LA	8	12.5	Thailand	[31]
	ELISA + MAT	6	83.3	Shanghai, China	[106]
Fishing cat (*Prionailurus viverrinus*)	IB + IFAT	40	55.0	Europe	Present study
	IFAT	1	0.00	Brazil	[29]
	DT	1	100.0	Thailand	[107]
	MAT	4	25.0	Midwestern USA	[11]
	MAT	4	50.0	Australia	[105]
	IFAT	1	0.00	California, USA	[32]
	LA	27	22.2	Thailand	[31]
Pallas’ cat (*Otocolobus manul*)	IB + IFAT	52	90.4	Europe	Present study
	DAT + IFAT	8	100.0	Austria	[19]
	EIA + LA	9	100.0	USA	[15]
	MAT	5	20.0	Midwestern USA	[11]
	MAT	3	66.7	Wisconsin, USA	[24]
	LA	4	100.0	Denver, USA	[17]
	ELISA	6	100.0	Oklahoma, USA	[22]
	IHA	3	100.0	California, USA	[23]
	IFAT	2	100.0	Czech Republic/Slovak Republic	[7]
	ELISA	14	78.6	Ohio, USA	[18]
Sand cat (*Felis margarita*)	IB + IFAT	87	54.0	Europe	Present study
	MAT	1	100.0	France	[14]
	MAT	20	70.0	UAE	[16]
	MAT	6	100.0	UAE	[28]

*Abbreviations*: IB, immunoblot; IFAT, immunofluorescent antibody test; DT, dye test; MAT, modified agglutination test; LA, latex agglutination test; DAT, direct agglutination test; EIA, enzyme immunoassay; ELISA, enzyme-linked immunosorbent assay; IHA, indirect hemagglutination, DNA, deoxyribonucleic acid

Differences in the susceptibility of various feline species for *T. gondii* have been discussed in previous studies; especially in Pallas’s cats, but also in sand cats, a high susceptibility to *T. gondii* has been assumed [15, 16]. Vertical transmission from an infected mother to kittens is likely to occur in these species [16, 19, 23, 28], which is also known to occur in domestic cats [61]. In other species, e.g. mice, there is a remarkable difference in susceptibility to oral infection with *T. gondii* among different inbred strains [62]. It might be possible that genetic factors also predispose some feline species for *T. gondii* infections.

In Pallas’s cats, an immunodeficiency (congenital or acquired) similar to FIV has been suspected to increase susceptibility [22]. FIV was found in captive and wild Pallas’s cats showing a unique monophyletic lineage of the virus in the population [63, 64]. Brown et al. [15] compared the general health status and indicators for chronic stress (corticoid metabolite measurement in fecal samples) of captive and wild Pallas’s cats and found similar results in both populations. In contrast to populations in human care, low percentages (13%) of *T. gondii* seropositive animals were found in the wild [15, 25]. Brown et al. [15] concluded that Pallas’s cats might not have co-evolved with *T. gondii* leading to a certain susceptibility for the parasite.

### Evaluation of potential risk factors for *T. gondii* seropositivity in zoos

Among 311 individuals included in the analysis, only 111 had been kept in a single institution until the time of sampling. As individuals that were transferred to other zoos (e.g. to enable breeding with a partner chosen by the stud book coordinator) were usually transported at young age and had spent most of their lifetime in the institution where they were sampled, it was assumed that zoo-specific conditions of the actual institution might have contributed to the risk of infection with *T. gondii* and seropositivity.

A first multilevel analysis in a generalized linear mixed model, in which “Zoo” was included as a random effects variable, showed that the age (in years) of the felids was strongly associated with the probability of testing seropositive for *T. gondii*. There are many reports demonstrating that older felids have a higher risk of testing positive than younger animals [8, 27, 65, 66]. This has been explained by the cumulative effect of the periods of potential exposure to the parasite during the lifetime of the animals. Differences in the mean age might have been a reason for the variability of *T. gondii* seroprevalences between different feline species included in the present study. For instance, only a few of the sampled black-footed cats were older than five years (26.7% seroprevalence). By contrast, most Pallas’s cats and rusty-spotted cats sampled were older than five years and these two species showed relatively high seroprevalences of 90.4% and 96.4%, respectively.

The final linear mixed model consisted of five variables including “Age”, feeding “Cattle” tissues, “Outdoor housing fenced in on all sides”, “Mesh size” of enclosures and “Wearing gloves” (Table 5). In this model, the probability of seropositivity increased statistically significantly with “Age”. Feeding “Cattle: frozen” relative to “Cattle: fresh”, “Outdoor housing fenced in on all sides: yes”, “Mesh size 2–5 cm” relative to “Mesh size > 5 cm” and “Wearing gloves: yes” had statistically significant protective effects.

In accordance with the bivariable analyses including the age of the animals as effect-modifying explanatory variable, the final model suggested that feeding cattle tissues that were frozen previously instead of fresh beef can protect felids from *T. gondii* infection, and thus from becoming *T. gondii-*seropositive. It has to be mentioned that although viable tissue cysts in cattle are probably rare [67], eating undercooked beef has been associated with human *T. gondii* infection. This might be due to larger amounts of cattle meat consumed, compared to meat of other species [68]. It has been estimated that 68% of meat-borne infections in humans in the Netherlands are due to beef products [69].

It can be suspected that feeding tissues from ruminants in fresh condition in general would pose a greater risk for *T. gondii-*seropositivity. The prevalence of *T. gondii* infection in sheep is considerably high in most European countries and worldwide [27, 70]. However, in the present study, very few zoos reported feeding the meat of small ruminants to its felids. This may be the reason why feeding tissues from sheep did not emerge as a relevant factor in our study.

The effect of freezing on the infectivity of *T. gondii* tissue cysts in pork has been studied and recommendations have been given to keep meat stored frozen to reach an internal temperature of – 12 °C for at least seven days prior to use to destroy tissue cysts [14, 27, 34–36]. Thus, the protective effects of freezing in the present study are in accordance with these findings. Other studies also recommended freezing meat prior to feeding in zoos to reduce the risk of exposure to *T. gondii* in carnivores [11, 14, 71, 72]. Silva et al. [39] hypothesized that feeding unfrozen meat in general poses a higher risk for exposure to *T. gondii* in felids.

In addition to feeding previously frozen cattle tissues, which appeared in the final model as a protective variable, bivariable analysis (including always the age of the animal) revealed that feeding carcasses or tissues from mice, rodents, ruminants or fowl after freezing also had a statistically significant protective effect. Feeding any of these items fresh represented the reference in these analyses and seemed to be a risk factor for seropositivity. A protective effect could also be seen when carcasses of mice were fed to felids either fresh or previously frozen (relative to the reference feeding mice fresh).

The prevalence of *T. gondii* in rodents and fowl produced for animal food is unknown, but they are likely to pose a low risk of transmitting *T. gondii*. Fowl or domestic small rodents are less likely to contain tissue cysts than pigs or small ruminants [70, 73, 74]. Despite the fact that commercial animal food suppliers mainly offer rats, mice or fowl frozen, especially small rodents like mice and rats used for animal food are produced under laboratory-like conditions. Commercial chickens are often raised indoors. In both laboratory or indoor-raised rodents or fowl, the risk for an exposure to *T. gondii* oocysts or tissue cysts can be considered as limited. Chickens kept indoors proved to have low prevalence of *T. gondii* infection compared to backyard chickens [75]. Overall, it can be assumed that the feeding of commercial fowl and rodents from animal food suppliers provides a high degree of biosafety.

The final model demonstrated that keeping felids in outdoor enclosures fenced in on all sides had a statistically significant protective effect against mounting an antibody response to *T. gondii*. This may be explained by a lower risk of wild animals entering the enclosure compared to enclosures with an open top, to which avian wildlife in particular might have easier access. Other authors reported that birds and small mammals might serve as a source of infection with *T. gondii* in feline enclosures. They may either serve as transport hosts (which might also include insects) [76–79] introducing oocysts to the exhibited felids or as intermediate hosts, i.e. infected pray species, thus exposing wild felids in human care to *T. gondii* tissue cysts [10, 11, 14, 71, 72, 80]. In general, it has been shown that cats showing preying behavior are more likely to be infected with *T. gondii* than cats that do not prey [81–83].

Mesh size was another factor, included in both the bivariable analysis and the final multivariable linear mixed model. Our statistical analysis suggests that keeping felids in enclosures with a mesh size of 2–5 cm had a statistically significant protective effect against developing antibodies to *T. gondii* as compared with mesh sizes above 5 cm as the reference. This result may be explained by the reduction of the number of rodents or other wild animals (i.e. possible intermediate hosts of *T. gondii*) that is able to enter the enclosures, although a mesh size of less than 5 cm is not considered as rodent-proof. However, a low mesh size may reduce the number of prey animals that can enter the enclosure. Not only rodents, but also other intermediate hosts such as avian wildlife, may play an important role in the transmission of *T. gondii* [84]. It therefore appears plausible that small mesh sizes (e.g. below 5 cm) may still have a protective effect against *T. gondii* infection, at least to a certain level.

Finally, the use of gloves for hygienic reasons had a statistically significant protective effect against *T. gondii-*seropositivity in the final linear mixed model. Wearing gloves might coincide with effective hand hygiene. This may reduce the probability of an accidental transmission of diseases including *T. gondii*. Moving contaminated items into the enclosure may lead to a contamination of non-protected hands with oocysts, which could in turn cause a secondary contamination e.g. of food preparations if these are handled without washing or protecting hands with gloves. In general, the route of transmission *via* contaminated hands is possible, but does not seem to be very likely. In addition, it may be assumed that institutions which ensure that their staff use gloves in their daily routines are generally more considerate about hygiene issues.

While variables that appeared to have statistically significant effects in the final model, bivariable analysis (including “Age” as effect-modifying and “Zoo” as random effects variable) indicated that also some other variables might have an effect on *T. gondii-*seropositivity. Although, the impact or the biological plausibility of the effect seem to be questionable for some of them (Table 4), the variables that had tested statistically significant (*P* < 0.1) in the bivariable analysis are discussed below.

Bivariable analysis suggested that felids from zoos, in which few litters (1–2 within one year, or 1–9 within five years) or more than three litters had been born during the last year, had a statistically significantly higher risk of testing serologically positive for antibodies to *T. gondii* as compared to felids from zoos with no litters born during the past year or the past five years. There are at least two possible explanations for this phenomenon. First, pregnancy may have an immunosuppressive effect on felids [85] and it can be hypothesized that this may have increased the risk of re-shedding *T. gondii* oocysts. However, in domestic cats re-shedding has so far only been reported under immunosuppressive corticoid treatment [86], but not yet during pregnancy. Another possible explanation is based on the observation that young felids seem to be more susceptible for intestinal infection and subsequent oocyst shedding than older felids. In older felids, immunity that has developed after a prior infection may prevent subsequent infections and thus renewed oocyst shedding [87, 88]. In addition, the intensity of oocyst shedding after a secondary infection is reduced compared to the shedding after the primary infection [86]. Therefore, oocyst contaminations may occur more often and might be more intense in zoos with litters of young and thus more susceptible felids than in zoos without young felids.

Little is known about potential differences in the capability of various felid species to shed *T. gondii* oocysts. Some studies demonstrated that oocyst shedding occurred in exotic felids, although oocyst production was not as efficient as in domestic cats [89, 90]. Shedding of oocysts in several episodes without signs of clinical disease were confirmed in wild cats (*Felis silvestris*) and Amur leopard cats (*Prionailurus bengalensis euptilura*) [91]. In Pallas’s cats, oocyst shedding coincided with clinical disease in juvenile individuals in addition to recurrent shedding of the infected queen [19]. It is not known if sand cats are able to excrete oocysts.

Keeping NWM close to felids protected the felids under examination statistically significantly against the development of a *T. gondii-*seropositive result. It is difficult to explain this observation by the biology of the parasite. Most probably, the protective effect observed for this variable is a confounder: Zoo staff members know about the increased risk of NWM becoming infected with *T. gondii* [92–96]. This might increase the awareness for hygienic measures around the NWM and feline enclosures, because felids are the final hosts for *T. gondii*, and thus explain the protective outcome.

Performing rabies vaccinations and a low deworming frequency (long deworming interval) appeared to have had a statistically significant protective effect and seemed to prevent wild felids in human care to test serologically *T. gondii-*positive. It is difficult to think of any biologically plausible explanation for these effects. It is therefore possible that these associations are spurious, which may be explained by the limitations of statistical modeling. Most likely, these variables represent confounders.

## Conclusions

A seroepidemiological study was conducted in 51 zoos in Europe and the Middle East. After validation in domestic cats, the reduviid bug *D. maxima* was used for blood sample collection in most of the 336 exotic cats of 17 different species. Study results clearly highlight that wild felids in human care (also including endangered species) in European and Middle Eastern institutions are widely exposed to *T. gondii*. A subsequent risk factor analysis including data of 311 small exotic cats of 10 species revealed that feeding previously frozen tissues, keeping animals housed fenced in on all sides and in enclosures with mesh sizes of 2–5 cm as well as wearing gloves when working inside enclosures seem to be the most relevant protective measures to prevent *T. gondii-*seropositivity in wild felids in human care. Further studies in small exotic cats are necessary to examine the influence of toxoplasmosis on population stability within breeding populations in human care.

## Supplementary information

**Additional file 1: Figure S1.** Questionnaire used to assess risk factors in zoos.

**Additional file 2: Table S1.** *Toxoplasma gondii*-specific antibodies as determined by immunoblot (IB) and immuno-fluorescent antibody test (IFAT).

**Additional file 3: Table S2.** Individual animal data.

**Additional file 4: Table S3.** Zoo data on putative risk factors.

**Additional file 5: Table S4.** Description of variables used for statistical analysis as determined from the questionnaire and from individual data using ZIMS (provided in Additional file 3: Table S2 and Additional file 4: Table S3).

**Additional file 6: Table S5.** Serological results in wild felids in human care stratified by zoo- and individual-related variables.

**Additional file 7: Table S6.** Results of factor analysis 1. **Table S7.** Results of factor analysis 2. **Table S8.** Results of factor analysis 3.

## Data Availability

All data generated or analyzed during the present study are included in this published article and its additional files.

## Abbreviations

AIC: Akaike information criterion
CITES: Convention on International Trade in Endangered Species of Wild Fauna and Flora
DAT: direct agglutination test
Dm0: blood from *Dipetalogaster maxima* at point 0
Dm1: blood from of *Dipetalogaster maxima* at point 1 (after 1 hr)
EAZA: European Association of Zoos and Aquaria
EEP: European Endangered species Programme
EIA: enzyme immunoassay
ELISA: enzyme-linked immunosorbent assay
ESB: European Studbook
FeLV: feline leukemia virus
FHV1: feline herpes virus 1
FIP: feline infectious peritonitis
FIV: feline immunodeficiency virus
IB: immunoblot
IFAT: immunofluorescent antibody test
IHA: indirect hemagglutination
L1–L5: larval instars 1–5
MAT: modified agglutination test
NWM: New World monkeys
PBS: phosphate-buffered saline
PVDF: polyvinylidene difluoride
SDS-PAGE: sodium dodecyl sulfate-polyacrylamide gel electrophoresis
Sig.: Significance
TAG: Taxon Advisory Group
TgSAG1: *T. gondii* tachyzoite surface antigen 1
V: conventional venous blood sample
*z*-value: standard score (regression coefficient divided by its standard error)
ZIMS: Zoological Information Management System
