# Supplementary material for: Toxoplasma gondii in small exotic felids from zoos in Europe and the Middle East: serological prevalence and risk factors
Source: Parasit Vectors. 2019 Sep 11;12:449. doi: 10.1186/s13071-019-3706-2 (PMC6737647; doi:10.1186/s13071-019-3706-2)
Supplement: Supplementary file 1 — Additional file 1: Figure S1. Questionnaire used to assess risk factors in zoos. [file 13071_2019_3706_MOESM1_ESM.pdf]

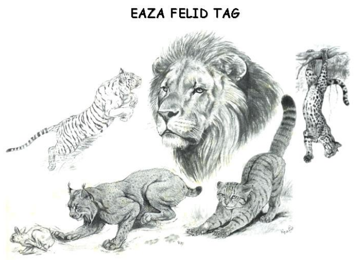

<u>Questionnaire on toxoplasmosis in small exotic felids</u>

Address of your Institution:

____

____

____

Name, Email and telephone number of the person answering the questionnaire:

____

____

Date: ____

How many small felids do you have currently in your zoo?

Species	Number of Individuals<br>Juvenile (m,f,x)	Adult (m,f,x)
Sand cat *(Felis margarita)*		
Black footed cat *(Felis nigripes)*		
Pallas cat *(Felis manul)*		
Fishing cat *(Prionailurus viverrinus)*		
Rusty-spotted cat *(Prionailurus rubiginosus phillipsi)*		
Geoffroy's cat *(Leopardus geoffroyi)*		
Oncilla *(Leopardus tigrinus)*		
Margay *(Leopardus wiedii)*		

m: male, f: female, x: sex unknown (i.e.: Sand cat 0,0,2 3,1,0)

**Feeding:**			
1. What do you feed your cats with?<br>(Multiple answers possible)	Mice	Frozen<br>regularly □ rarely □	Fresh<br>regularly □ rarely □
	Rats	Frozen<br>regularly □ rarely □	Fresh<br>regularly □ rarely □
	Sheep/ Goat	Frozen<br>regularly □ rarely □	Fresh<br>regularly □ rarely □
	Cattle	Frozen<br>regularly □ rarely □	Fresh<br>regularly □ rarely □
	Horse	Frozen<br>regularly □ rarely □	Fresh<br>regularly □ rarely □
	Fowl/ chicks	Frozen<br>regularly □ rarely □	Fresh<br>regularly □ rarely □
	Fish	Frozen<br>regularly □ rarely □	Fresh<br>regularly □ rarely □
	Fruit/ Vegetables		regularly □ rarely □
	Catfood	Canned<br>regularly □ rarely □	Dry<br>regularly □ rarely □

Questionnaire on toxoplasmosis in small exotic felids

2. Where do you get the food (meat) from?		
3. What is the temperature of your food storage?	10°C to 5°C	□
	5°C to 0°C	□
	0°C to -5°C	□
	-5°C to -10°C	□
	-10°C to -15°C	□
	-15°C to -20°C	□
	Under -20°C	□
4. Is the preparation of meat and fruits/vegetables separated?	No	□
	Preparation in different rooms	□
	Separate areas in the kitchen	□
	Separate cutting boards in use	□
5. How long is the food frozen before feeding?	Less than 1 week	□
	1-2 weeks	□
	More than 2 weeks	□
6. Where does the drinking water come from?	Rain water	□
	Well water	□
	Tap water	□
	Bottled water	□
7. How often do you clean the water bowl?	Daily	□
	Every other day	□
	Once a week	□
	We do not change the water	□

**Husbandry of small felids:**		
1. How often do you remove feces in the enclosures?	Daily	□
	Every second day	□
	Once a week	□
	More infrequently	□
2. Is there a litter box?	Yes □	No □
3. Are any of these animals kept close by? (within 50 meters)	Other felids Yes □	No □
	New world monkeys Yes □	No □
	Marsupials Yes □	No □
4. Do the same zookeepers care for more than one cat species?	Yes □	No □
5. Do the same zookeepers care for cats and Marsupials/ New world monkeys?	Yes □	No □
6. Do you use hygiene devices to avoid spreading of pathogenic agents?	Yes □	No □

EAZA FELID TAG

Questionnaire on toxoplasmosis in small exotic felids

7. If you do, what kind of hygiene devices? (Multiple answers possible)	Hand wash facilities at all enclosures	□
	Hand disinfection at all enclosures	□
	Disinfective footbath between enclosures	□
	Protective clothing: *Disposable gloves*	□
	Disposable shoe covers	□
	Disposable suits	□
8. What kind of bedding material do you use in your enclosures? (Multiple answers possible)	Shavings	□
	Hay	□
	Straw	□
	Sand	□
	Other	□
9. How often do you change the bedding material?	Daily	□
	Weekly	□
	Monthly	□
	More infrequently	□
10. Do the cats get any items for enrichment?	Yes □	No □
11. If yes, what kind of items for enrichment? (Multiple answers possible)	Branches etc. out of nature	□
	Industrial cat toys (Balls…)	□
	Other (Paperbags…)	□
12. What kind of enclosures do you have?	In a house, no contact to wildlife possible	□
	Outdoors fenced in on all sides (also from above), indirect contact to wildlife possible	□
	Enclosure open from above, direct contact to wildlife possible	□
13. Total size of the enclosure	$< 20m^2$	□
	$20\text{-}50m^2$	□
	$>50m^2$	□
14. What mesh size does the fence of your Enclosures have?	≤ 1cm	□
	1-2 cm	□
	2-5 cm	□
	> 5 cm	□
15. Do damaged fences allow access for mice?	Yes □	No □

**Pest control:**		
1. Do you do any pest control?	Yes □	No □
2. If yes, how do you do pest control?	Traps	□
	Poison	□
	Domestic cats	□
3. How often?	Once a month	□
	Quarterly	□
	Biannual	□
	Once a Year	□
4. Do you have stray cats at the zoo?	Yes □	No □

EAZA FELID TAG

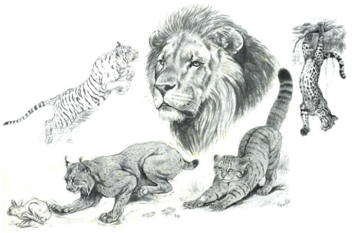

Questionnaire on toxoplasmosis in small exotic felids

5. If yes, how many?			
**Toxoplasmosis:**			
1. How many deaths did you have the last 5 years?	Species	No. of death<br>Juvenile (m,f,x)	Adult (m,f,x)
	Sand cat *(Felis margarita)*		
	Black footed cat *(Felis nigripes)*		
	Pallas cat *(Felis manul)*		
	Fishing cat *(Prionailurus viverrinus)*		
	Rusty-spotted cat *(Prionailurus rubiginosus phillipsi)*		
	Geoffroy's cat *(Leopardus geoffroyi)*		
	Oncilla *(Leopardus tigrinus)*		
	Margay *(Leopardus wiedii)*		

2. Did you in the last 5 years look for *T. gondii* or antibodies against *Toxoplasma* in any animals (felids and non felids)?				Yes □	No □
3. If yes, please give details:					
Species	No. of individuals	Test used and results	In which context did you look for it?	*T. gondii* suspected cause of death?	
				Yes □	No □
				Yes □	No □
				Yes □	No □
				Yes □	No □
				Yes □	No □
				Yes □	No □
				Yes □	No □
				Yes □	No □
				Yes □	No □
				Yes □	No □
4. Did you treat any of your animals against toxoplasmosis?				Yes □	No □
5. If yes, what treatment did you use?					
6. Was the treatment successful?				Yes □	No □

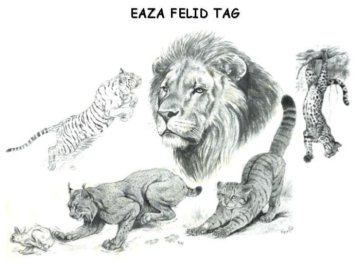

Questionnaire on toxoplasmosis in small exotic felids

**General veterinary treatment of small felids:**		
1. Are the animals vaccinated?	Yes □	No □
2. If yes, what do you vaccinate for?	Cat flu	□
	Parvovirus	□
	Rabies	□
	Leucosis	□
	FIP	□
3. Do the cats get regularly dewormed?	Yes □	No □
4. If yes, how often?	Every 3 months	□
	Half a year	□
	Once a year	□
	More infrequently	□
5. Do you regularly check for parasites in the feces?	Yes □	No □
6. If yes, how often?	Every 3 months	□
	Half a year	□
	Once a year	□
	More infrequently	□

7. Was any of the following disease diagnosed in any of your small felids during the last 5 years?		
Disease	Species infected (number of animals)	What kind of test did you use?
**FHV1** (Feline Herpes virus 1)		
**Calicivirus**		
**FeLV** (Feline leukemia virus)		
**FIP** (Corona virus)		
**FIV** (Feline immunedeficiency virus)		
**Feline Distemper** (Feline panleuko-penia virus)		
