# Supplementary material for: Toxoplasma gondii in small exotic felids from zoos in Europe and the Middle East: serological prevalence and risk factors
Source: Parasit Vectors. 2019 Sep 11;12:449. doi: 10.1186/s13071-019-3706-2 (PMC6737647; doi:10.1186/s13071-019-3706-2)
Supplement: Supplementary file 2 — Additional file 2: Table S1. Toxoplasma gondii-specific antibodies as determined by immunoblot (IB) and immuno-fluorescent antibody test (IFAT). [file 13071_2019_3706_MOESM2_ESM.docx]

**Additional file 2: Table S1** *T. gondii*-specific antibodies as determined by immunoblot (IB) and immunofluorescent antibody test (IFAT). Reciprocal IFAT titer in venous blood (Titer V) and in bug-derived plasma taken immediately (Titer Dm0) and one hour (Titer Dm1) after engorgement. Results highlighted in grey were positive (IFAT cut-off in plasma: 100). The “NA” indicates that there was no sample available.

| **Sample No.** | **IB** | **IFAT titer** | | |
| --- | --- | --- | --- | --- |
|  | **V** | **V** | **Dm0** | **Dm1** |
| 1 | Pos. | 1600 | 800 | 1600 |
| 2 | Pos. | 3200 | 3200 | 3200 |
| 3 | Pos. | 800 | 800 | 800 |
| 4 | Neg. | <25 | <25 | <25 |
| 5 | Pos. | 3200 | 6400 | 6400 |
| 6 | Neg. | <25 | <25 | <25 |
| 7 | Pos. | 800 | 1600 | 3200 |
| 8 | Neg. | 50 | 50 | 50 |
| 9 | Neg. | <25 | <25 | <25 |
| 10 | Pos. | 800 | 800 | 1600 |
| 11 | Neg. | <25 | <25 | <25 |
| 12 | Neg. | <25 | 25 | 25 |
| 13 | Neg. | <25 | <25 | <25 |
| 14 | Pos. | 6400 | 6400 | 12800 |
| 15 | Neg. | <25 | <25 | <25 |
| 16 | Neg. | <25 | <25 | <25 |
| 17 | Neg. | <25 | <25 | <25 |
| 18 | Neg. | 25 | 25 | 25 |
| 19 | Neg. | 25 | <25 | <25 |
| 20 | Neg. | <25 | <25 | NA |
| 21 | Pos. | 800 | 1600 | 1600 |
| 22 | Neg. | 25 | <25 | 25 |
| 23 | Neg. | 25 | NA | 50 |
| 24 | Neg. | <25 | 25 | 25 |
| 25 | Pos. | 1600 | 1600 | 1600 |
| 26 | Pos. | 800 | 800 | 800 |
| 27 | Neg. | <25 | <25 | <25 |
| 28 | Neg. | <25 | <25 | <25 |
| 29 | Pos. | 3200 | 3200 | 3200 |
| 30 | Neg. | <25 | <25 | <25 |
| 31 | Neg. | <25 | <25 | <25 |
| 32 | Neg. | <25 | <25 | <25 |
| 33 | Neg. | <25 | <25 | <25 |
| 34 | Neg. | <25 | <25 | <25 |
| 35 | Pos. | 3200 | 3200 | 3200 |
| 36 | Neg. | <25 | 25 | <25 |
| 37 | Pos. | 1600 | 1600 | 3200 |
| 38 | Neg. | <25 | 25 | <25 |
| 39 | Neg. | 25 | 25 | 25 |
| 40 | Pos. | 400 | 200 | 400 |
| 41 | Pos. | 1600 | 3200 | NA |
| 42 | Pos. | 12800 | 12800 | 12800 |
| 43 | Neg. | <25 | <25 | <25 |
| 44 | Pos. | 400 | 400 | 800 |
| 45 | Pos. | 800 | 800 | 1600 |
| 46 | Neg. | <25 | <25 | <25 |
| 47 | Neg. | <25 | <25 | <25 |
| 48 | Neg. | <25 | <25 | <25 |
| 49 | Pos. | 1600 | 1600 | 1600 |
| 50 | Pos. | 3200 | 3200 | 3200 |
| 51 | Neg. | <25 | <25 | <25 |
| 52 | Pos. | 6400 | 6400 | 12800 |
| 53 | Neg. | <25 | <25 | <25 |
| 54 | Neg. | <25 | <25 | <25 |
| 55 | Neg. | <25 | <25 | <25 |
| 56 | Neg. | <25 | <25 | 50 |
| 57 | Neg. | 25 | 50 | 50 |
| 58 | Pos. | 1600 | 1600 | 3200 |
| 59 | Pos. | 3200 | 3200 | 6400 |
| 60 | Pos. | 3200 | 3200 | 3200 |
| 61 | Neg. | <25 | <25 | <25 |
| 62 | Pos. | 3200 | 1600 | 3200 |
| 63 | Pos. | 3200 | 3200 | 3200 |
| 64 | Pos. | 3200 | 3200 | 3200 |
| 65 | Neg. | <25 | <25 | <25 |
| 66 | Pos. | 3200 | 3200 | 3200 |
| 67 | Neg. | 25 | 25 | 50 |
| 68 | Neg. | <25 | <25 | 25 |
| 69 | Neg. | 50 | 50 | 25 |
| 70 | Pos. | 800 | 800 | 800 |
