# Supplementary material for: Toxoplasma gondii in small exotic felids from zoos in Europe and the Middle East: serological prevalence and risk factors
Source: Parasit Vectors. 2019 Sep 11;12:449. doi: 10.1186/s13071-019-3706-2 (PMC6737647; doi:10.1186/s13071-019-3706-2)
Supplement: Supplementary file 5 — Additional file 5: Table S4. Description of variables used for statistical analysis as determined from the questionnaire and from individual data using ZIMS (provided in Additional file 3: Table S2 and Additional file 4: Table S3). [file 13071_2019_3706_MOESM5_ESM.docx]

**Additional file 5: Table S4.** Variables used in statistical analysis as obtained using a standard questionnaire or from ZIMS (Zoological Information Management System) and provided in Additional file 3: Table S2 and Additional file 4: Table S3.

| **Variable as determined for individual animals and as determined by questionnaire** | **Data source** | **Explanation** | **Categories** | **Modification of variables** |
| --- | --- | --- | --- | --- |
| Zoo | Zoo data, Animal data | Zoo named by the region |  |  |
| Species | Animal data | Name of the species sampled |  |  |
| SampleNo | Animal data | Sample identification |  |  |
| IFAT | Animal data | Titer determined by IFAT |  | IFAT_Categories, Serology (based on IFAT and immunoblot data) |
| FLIImmunoblot | Animal data | FLI Immunoblot result | “positive”, “negative”, “inconclusive” | Serology (based on IFAT and immunoblot data) |
| Age | Animal data | Date of sample collection |  | Age (based on days of birth and sampling) |
| Sex | Animal data |  | Male: “m”, female: “f” |  |
| Lived.in.1.Institution.only | Animal data | No transport to other Zoos until sample collection. | “0”: no, “1”: yes |  |
| Mice-fresh | NA | Fresh mice used as food items | “0”: no, “1”: yes | Feeding-Mice, Feeding-Rodents |
| Mice-frozen | NA | Frozen mice used as food items | “0”: no, “1”: yes |  |
| Mice | Zoo data | Based on “Mice” – “Frozen” or “Fresh” | “No”, “Fresh”, “Frozen”, “FreshFrozen” |  |
| Rat-fresh | NA | Fresh rats used as food items | “0”: no, “1”: yes | Feeding-Rats, Feeding-Rodents |
| Rat-frozen | NA | Frozen rats used as food items | “0”: no, “1”: yes |  |
| Rats | Zoo data | Based on “Rats” – “Frozen” or “Fresh” | “No”, “Fresh”, “Frozen”, “FreshFrozen” |  |
| Rodents | Zoo data | Based on „Rats“ or „Mice“ – „Frozen“ or „Fresh“ | “No”, “Fresh”, “Frozen”, “FreshFrozen” |  |
| Sheep-fresh | NA | Fresh sheep used for feeding | “0”: no, “1”: yes | Feeding-Sheep, Feeding-Ruminants, Feeding-Ungulates |
| Sheep-frozen | NA | Frozen sheep used for feeding | “0”: no, “1”: yes |  |
| Sheep | Zoo data | Based on “Sheep” – “Frozen” or “Fresh” | “No”, “Fresh”, “Frozen”, “FreshFrozen” |  |
| Cattle-fresh | NA | Fresh cattle used for feeding | “0”: no, “1”: yes | Feeding-Cattle, Feeding-Ruminants, Feeding-Ungulates |
| Cattle-frozen | NA | Frozen cattle used for feeding | “0”: no, “1”: yes |  |
| Cattle | Zoo data | Based on “Cattle” – “Frozen” or “Fresh” | “No”, “Fresh”, “Frozen”, “FreshFrozen” |  |
| Ruminants | Zoo data | Based on „Sheep“ or „Cattle“ – „Frozen“ or „Fresh“ | “No”, “Fresh”, “Frozen”, “FreshFrozen” |  |
| Horse-fresh | NA | Fresh horse used for feeding | “0”: no, “1”: yes | Feeding-Horses, Feeding-Ungulates |
| Horse-frozen | NA | Frozen horse used for feeding | “0”: no, “1”: yes |  |
| Horses | Zoo data | Based on “Horses” – „Frozen“ or „Fresh“ | “No”, “Fresh”, “Frozen”, “FreshFrozen” |  |
| Ungulates | Zoo data | Based on „Sheep“ or „Cattle“ or “Horses” – „Frozen“ or „Fresh“ | “No”, “Fresh”, “Frozen”, “FreshFrozen” |  |
| Fowl-fresh | NA | Fresh fowl used for feeding | “0”: no, “1”: yes | Feeding-Fowl |
| Fowl-frozen | NA | Frozen fowl used for feeding | “0”: no, “1”: yes |  |
| Fowl | Zoo data | Based on “Fowl” – „frozen“ or „fresh“ | “No”, “Fresh”, “Frozen”, “FreshFrozen” |  |
| Fish-fresh | NA | Fresh fish used for feeding | “0”: no, “1”: yes | Feeding-Fish |
| Fish-frozen | NA | Frozen fish used for feeding | “0”: no, “1”: yes |  |
| Fish | Zoo data | Based on “Fish” – „frozen“ or „fresh“ | “No”, “Fresh”, “Frozen”, “FreshFrozen” |  |
| Fruit-vegetables | NA | Fruits and vegetables used for feeding | “0”: no, “1”: yes | Feeding-Fruit |
| Fruit | Zoo data | Based on “Fruits-vegetables” | „Yes“, „No“ |  |
| Catfood-cannded | NA | Feeding canned cat food | “0”: no, “1”: yes | Feeding-Catfood |
| Catfood-dry | NA | Feeding dry cat food | “0”: no, “1”: yes |  |
| Catfood | Zoo data | Based on “Cat-food canned” and “Catfood-dry” | „Dry“, „CannedDry“, „Canned“ |  |
| Foodstorage<-20°C | NA | Temperature of food storage <-20°C | “0”: no, “1”: yes | FoodStorage |
| Foodstorage-20°C- -15°C | NA | Temperature of food storage -20°C- -15°C | “0”: no, “1”: yes |  |
| Foodstorage-15°C- -10°C | NA | Temperature of food storage -15°C- -10°C | “0”: no, “1”: yes |  |
| Foodstorage-10°C- -5°C | NA | Temperature of food storage -10°C- -5°C | “0”: no, “1”: yes |  |
| Foodstorage-5°C-0°C | NA | Temperature of food storage -5°C-0°C | “0”: no, “1”: yes |  |
| Foodstorage0°C-5°C | NA | Temperature of food storage 0°C-5°C | “0”: no, “1”: yes |  |
| Foodstorage5°C-10°C | NA | Temperature of food storage 5°C-10°C | “0”: no, “1”: yes |  |
| FoodStorage | Zoo data | Based on “Foodstorage….” | „Cool“, „CoolFrozen“, „Frozen“ |  |
| Preparation-rooms | NA | Preparation of meat and fruits/vegetables in different rooms | “0”: no, “1”: yes | MeatFruitSeperation |
| Preparation-areas | NA | Preparation of meat and fruits/vegetables in different areas | “0”: no, “1”: yes |  |
| Preparation-board | NA | Preparation of meat and fruits/ vegetables on separate cutting boards | “0”: no, “1”: yes |  |
| Preparation-noseparation | NA | No separate preparation of meat and fruits/vegetables | “0”: no, “1”: yes |  |
| MeatFruitSeparation | Zoo data | Based on „Preparation-…“ | „No“, „SeparateByProcess“, „SeparateByRooms“ |  |
| FoodFrozen-<1w | NA | Freezing meat for less than 1 week | “0”: no, “1”: yes | FoodFrozen |
| FoodFrozen-1-2w | NA | Freezing meat for 1-2 weeks | “0”: no, “1”: yes |  |
| FoodFrozen->2w | NA | Freezing meat for more than two weeks | “0”: no, “1”: yes |  |
| FoodFrozen | Zoo data | Based on „FoodFrozen-…“ | „Sometines<1Week“, „OneWeekAtLeast, More>2Weeks“ |  |
| Rainwater | NA | Rain water as main water source | “0”: no, “1”: yes | Water |
| Tapwater | NA | Tap water as main water source | “0”: no, “1”: yes |  |
| Wellwater | NA | Well water as main water source | “0”: no, “1”: yes |  |
| Bottledwater | NA | Bottled water as main water source | “0”: no, “1”: yes |  |
| Water | Zoo data | Based on „Rainwater“, „Tapwater“, „Wellwater“, „Bottledwater“ | „Tap“, „Well“, „TapWell“, „RainTap“ |  |
| Cleanwater-Daily | NA | Daily water renewal | “0”: no, “1”: yes | Cleanwater |
| Cleanwater-2ndday | NA | Every other day water renewal | “0”: no, “1”: yes |  |
| Cleanwater-Weekly | NA | Once a week water renewal | “0”: no, “1”: yes |  |
| Cleanwater-no | NA | No water change | “0”: no, “1”: yes |  |
| Cleanewater | Zoo data | Based on „Cleanwater-…“ | „noChange“, „daily“, „weekly“, „dailyWeekly“, every2ndDay“ |  |
| FecesRemoval-Daily | NA | Daily feces removal | “0”: no, “1”: yes | FecesRemoval |
| FecesRemoval-2ndday | NA | Feces removal every second day | “0”: no, “1”: yes |  |
| FecesRemoval-Weekly | NA | Weekly feces removal | “0”: no, “1”: yes |  |
| FecesRemoval-Infreq | NA | Infrequent feces removal | “0”: no, “1”: yes |  |
| FecesRemoval | Zoo data | Based on „FecesRemoval-…“ | „Daily“, „NotDaily“ |  |
| Litterbox | Zoo data | Availability of a litterbox | “0”: no, “1”: yes |  |
| FelidsCloseby | Zoo data | Other felids kept close by (within 50m) | “0”: no, “1”: yes |  |
| MarsupialsCloseby | Zoo data | Marsupials kept close by (within 50m) | “0”: no, “1”: yes |  |
| NWMCloseby | Zoo data | New World monkeys kept close by (within 50m) | “0”: no, “1”: yes |  |
| CareOtherCats | Zoo data | Animal attendants look also after other cat species | “0”: no, “1”: yes |  |
| CareCatsPlusNWM_Marsupials | Zoo data | Animal attendants look also after Marsupials and/or New World Monkeys | “0”: no, “1”: yes |  |
| Hygiene | Zoo data | Availability of hygienic devices | “0”: no, “1”: yes |  |
| HandWashing | Zoo data | Availability of hand wash facilities at all enclosures | “0”: no, “1”: yes |  |
| HandDisinfection | Zoo data | Availability of hand disinfection at all enclosures | “0”: no, “1”: yes |  |
| FeetDisinfection | Zoo data | Availability of disinfective footbath between enclosures | “0”: no, “1”: yes |  |
| ShoeCovers | Zoo data | Availability of disposable shoe covers | “0”: no, “1”: yes |  |
| Using gloves | Zoo data | Availability of disposable gloves | “0”: no, “1”: yes |  |
| ShavingsBeddingMaterial | Zoo data | Wood shavings as bedding material | “0”: no, “1”: yes |  |
| HayBeddingMaterial | Zoo data | Hay as bedding material | “0”: no, “1”: yes |  |
| StrawBeddingMaterial | Zoo data | Straw as bedding material | “0”: no, “1”: yes |  |
| SandBeddingMaterial | Zoo data | Sand as bedding material | “0”: no, “1”: yes |  |
| OtherBeddingMaterial | Zoo data | Other bedding material | “0”: no, “1”: yes | BeddingChange |
| BeddingChange-Daily | NA | Daily change of bedding material | “0”: no, “1”: yes |  |
| BeddingChange-Weekly | NA | Weekly change of bedding material | “0”: no, “1”: yes |  |
| BeddingChange-Monthly | NA | Monthly change of bedding material | “0”: no, “1”: yes |  |
| BeddingChange-Infreq | NA | Infrequent change of bedding material | “0”: no, “1”: yes |  |
| BeddingChange | Zoo data | Based on „Bedding-…“ | „Not“, „Infreq“, „Month“, Week“, „Daily“ |  |
| Enrichment | Zoo data | General use of items for enrichment | “0”: no, “1”: yes |  |
| BranchesEnrichment | Zoo data | Use of natural materials like branches for enrichment | “0”: no, “1”: yes |  |
| IndustrialToysEnrichment | Zoo data | Use of industrial toys for enrichment | “0”: no, “1”: yes |  |
| OtherEnrichment | Zoo data | Use of other items for enrichment (paper bags…) | “0”: no, “1”: yes |  |
| Indoor | Zoo data | Housing indoors, no contact to wildlife possible | “0”: no, “1”: yes |  |
| Outdoor_Fencedin-allsides | Zoo data | Outdoors fenced in on all sides (also from above), indirect contact to wildlife possible | “0”: no, “1”: yes |  |
| Outdoor_Opentop | Zoo data | Enclosure open from above, direct contact to wildlife possible | “0”: no, “1”: yes |  |
| SizeSmaller20 | Zoo data | Enclosure size < 20 m^2^ | “0”: no, “1”: yes |  |
| Size20Until50 | Zoo data | Enclosure size 20-50 m^2^ | “0”: no, “1”: yes |  |
| SizeLarger50 | Zoo data | Enclosure size >50 m^2^ | “0”: no, “1”: yes | MeshSize |
| MeshSize1-2 | NA | Mesh size 1cm-2 cm | “0”: no, “1”: yes |  |
| MeshSize2-5 | NA | Mesh size 2 cm-5 cm | “0”: no, “1”: yes |  |
| MeshSize>5 | NA | Mesh size > 5 cm | “0”: no, “1”: yes |  |
| MeshSize | Zoo data | Based on “MeshSize…” | “MeSi2Unt5”=there are enclosures with a mesh size 2-5 cm; “MeSi1Unt2”=there are only enclosures with mesh sizes 1-2 cm; “MeSiGr5”=there are only enclosures with a mesh size > 5cm |  |
| PestControl | Zoo data | Performance of pest control in general | “0”: no, “1”: yes |  |
| PestControl-Cats | Zoo data | Cats used for pest control | “0”: no, “1”: yes |  |
| PestControl-Poison | Zoo data | Poison used for pest control | “0”: no, “1”: yes |  |
| PestControl-Traps | Zoo data | Traps used for pest control | “0”: no, “1”: yes | PestControl Frequency-Month |
| PestControl-Monthly | NA | Frequency of pest control: “Monthly” | “0”: no, “1”: yes |  |
| PestControl-Quarterly | NA | Frequency of pest control: “Quarterly” | “0”: no, “1”: yes |  |
| PestControl-Biannual | NA | Frequency of pest control: “Biannual” | “0”: no, “1”: yes |  |
| PestControl-Yearly | NA | Frequency of pest control: “Annually” | “0”: no, “1”: yes |  |
| PestControl Frequency-Month | Zoo data | Based on „PestControl…“ | „Infreq“, „0“, „0.25“, „1“, „2“, „3“, „6“, „12“ monthly intervals |  |
| StrayCats | Zoo data | Estimated number of stray cats at the compound |  |  |
| ToxoTest | Zoo data | Implementation of *T. gondii* tests in any animal species within 5 years prior to blood sampling. | “0”: no, “1”: yes |  |
| ToxoFound | Zoo data | Number of positive *T. gondii* tests in any animal species within 5 years prior to blood sampling. |  |  |
| Death | Zoo data | Occurrence of deaths among the felid species tested within 5 years prior to blood sampling | “0”: no, “1”: yes |  |
| NoDeath | Zoo data | Numbers of deaths among the felid species tested within 5 years prior to blood sampling |  |  |
| Vaccine | Zoo data | Implementation of vaccinations in general in small felids | “0”: no, “1”: yes |  |
| CatFlu | Zoo data | Implementation of vaccinations against cat flu in small felids | “0”: no, “1”: yes |  |
| Parvovirus | Zoo data | Implementation of parvovirus vaccinations in small felids | “0”: no, “1”: yes |  |
| Rabies | Zoo data | Implementation of rabies vaccinations in small felids | “0”: no, “1”: yes |  |
| Leucosis | Zoo data | Implementation of feline leucosis vaccinations in small felids | “0”: no, “1”: yes |  |
| FIP | Zoo data | Implementation of FIP vaccinations in small felids | “0”: no, “1”: yes |  |
| Deworming | Zoo data | General implementation of deworming | “0”: no, “1”: yes | DewormingFrequency-Month |
| DewormingInterval-Quarterly | NA | Deworming interval: Quarterly | “0”: no, “1”: yes |  |
| DewormingInterval-Biannually | NA | Deworming interval: Biannually | “0”: no, “1”: yes |  |
| DewormingInterval-Annually | NA | Deworming interval: Annually | “0”: no, “1”: yes |  |
| DewormingInterval-Infreq | NA | Infrequent deworming intervals | “0”: no, “1”: yes |  |
| DewormingInterval-Month | Zoo data | Based on “DewormingFrequency-…” | „3“, „4“, „6“, „12“ deworming intervals in months |  |
| Check | Zoo data | General implementation of parasite checks | “0”: no, “1”: yes | CheckFrequency-Month |
| CheckFrequency-Quarterly | NA | Interval for parasite checks: Quarterly | “0”: no, “1”: yes |  |
| CheckFrequency-Biannually | NA | Interval for parasite checks: Biannually | “0”: no, “1”: yes |  |
| CheckFrequency-Annually | NA | Interval for parasite checks: Annually | “0”: no, “1”: yes |  |
| CheckFrequency-Infreq | NA | Infrequent parasite check intervals | “0”: no, “1”: yes |  |
| CheckFrequency-Month | Zoo data |  |  |  |
| SourceFood | NA | Open question on the origin of food items used for the felids tested. Categorized after data collection. | “Laboratories”, “hunting”, “own production”, “local suppliers”, “slaughters”, “private breeder”, “commercial animal food supplier” | LabsSpecies, HuntingSpecies, OwnProductionSpecies, LocalSupplierSpecies, SlaughtersSpecies, PrivateBreederSpecies, CommercialProducerSpecies |
| LabsSpecies | Zoo data | Food items/species produced by laboratories | “Rats”, “RatsMice”, “RatsMiceFowl” |  |
| HuntingSpecies | Zoo data | Food items/species produced by hunting | “Rabbits”, “RabbitsSquirrelPigeon” |  |
| OwnProductionSpecies | Zoo data | Food items/species out of own production | “Fowl”, “GoatDeer”, “Mice”, “Rats”, “RatsMice” |  |
| LocalSupplierSpecies | Zoo data | Food items/species produced by local suppliers | “Cattle”, “HorseCattle” |  |
| SlaughtersSpecies | Zoo data | Food items/species produced by slaughters | “Fowl”, “Fish”, “FowlFish”, “MiceFish”, “MiceRats”, “RabbitsHorse” |  |
| PrivateBreederSpecies | Zoo data | Food items/species produced by private breeders | “Fowl”, “Horse”, “RabbitFowl” |  |
| CommercialProducerSpecies | Zoo data | Food items/species produced by commercial producers | “Fowl”, “FowlCatfood”, “FowlFish”, “Mice”, “Rabbits”, “Rats”, “RatsFowl”, “RatsFowlMice” |  |
| NoFelidsTotal | Zoo data | Number of felids (all species including small and big cats) as determined by ZIMS |  |  |
| No. Litters within 1 year | Zoo data | Number of litters born per zoo among all feline species within 1 year prior to sampling |  | LittersWithin1Year |
| LittersWithin1Year | Zoo data | Based on „LittersWithin1Year“ | “0“=0, “s“=1-2, “l“=≥3 |  |
| No. Litters within 5 years | Zoo data | Number of liters born per zoo among all feline species within 5 years prior to sampling |  | LittersWithin5Year |
| LittersWithin5Years | Zoo data | Based on „LittersWithin5Years“ | “0“=0, “s“=1-9, “l“=≥10 |  |
