# Supplementary material for: Toxoplasma gondii in small exotic felids from zoos in Europe and the Middle East: serological prevalence and risk factors
Source: Parasit Vectors. 2019 Sep 11;12:449. doi: 10.1186/s13071-019-3706-2 (PMC6737647; doi:10.1186/s13071-019-3706-2)
Supplement: Supplementary file 6 — Additional file 6: Table S5. Serological results in wild felids in human care stratified by zoo- and individual-related variables. [file 13071_2019_3706_MOESM6_ESM.docx]

**Additional file 6: Table S5** Serological results in wild felids in human care stratified by zoo- and individual animal-related variables

| **Variable** | **Variable category (No. of zoos)** | **Serological result** | | |
| --- | --- | --- | --- | --- |
|  |  | **Proportion of positives (%)** | **No. Positive** | **Total** |
| **Feeding mice** |  |  |  |  |
|  | Fresh (11) | 76.09 | 70 | 92 |
|  | Fresh or frozen (10) | 58.70 | 27 | 46 |
|  | Frozen (19) | 60.00 | 60 | 100 |
|  | No mice (8) | 62.96 | 17 | 27 |
|  | NA (2) | 47.83 | 22 | 46 |
|  | **Total (50)** | **63.02** | **196** | **311** |
| **Feeding rats** |  |  |  |  |
|  | Fresh (9) | 71.19 | 42 | 59 |
|  | Fresh or frozen (9) | 68.35 | 54 | 79 |
|  | Frozen (22) | 60.95 | 64 | 105 |
|  | No rats (8) | 63.64 | 14 | 22 |
|  | NA (2) | 47.83 | 22 | 46 |
|  | **Total (50)** | 63.02 | 196 | 311 |
| **Feeding cattle** |  |  |  |  |
|  | Fresh (6) | 76.19 | 16 | 21 |
|  | Fresh or frozen (5) | 69.86 | 51 | 73 |
|  | Frozen (14) | 45.83 | 22 | 48 |
|  | No cattle (23) | 69.11 | 85 | 123 |
|  | NA (2) | 47.83 | 22 | 46 |
|  | **Total (50)** | **63.02** | **196** | **311** |
| **Feeding sheep** |  |  |  |  |
|  | Fresh (5) | 82.76 | 24 | 29 |
|  | Fresh or frozen (1) | 64.52 | 20 | 31 |
|  | Frozen (2) | 00.00 | 0 | 3 |
|  | No sheep (40) | 64.36 | 130 | 202 |
|  | NA (2) | 47.83 | 22 | 46 |
|  | **Total (50)** | **63.02** | **196** | **311** |
| **Feeding horse** |  |  |  |  |
|  | Fresh (4) | 73.33 | 11 | 15 |
|  | Fresh or frozen (4) | 81.13 | 43 | 53 |
|  | Frozen(15) | 64.20 | 52 | 81 |
|  | No horse (25) | 58.62 | 68 | 116 |
|  | NA (2) | 47.83 | 22 | 46 |
|  | **Total (50)** | **63.02** | **196** | **311** |
| **Feeding fowl** |  |  |  |  |
|  | Fresh (7) | 86.21 | 25 | 29 |
|  | Fresh or frozen (12) | 66.15 | 86 | 130 |
|  | Frozen (27) | 57.45 | 54 | 94 |
|  | No fowl (2) | 75.00 | 9 | 12 |
|  | NA (2) | 47.83 | 22 | 46 |
|  | **Total (50)** | **63.02** | **196** | **311** |
| **Feeding fish** |  |  |  |  |
|  | Fresh (5) | 78.57 | 11 | 14 |
|  | Fresh or frozen (4) | 76.60 | 36 | 47 |
|  | Frozen (22) | 63.57 | 82 | 129 |
|  | No fish (17) | 60.00 | 45 | 75 |
|  | NA (2) | 47.83 | 22 | 46 |
|  | **Total (50)** | **63.02** | **196** | **311** |
| **Feeding fruit** |  |  |  |  |
|  | No fruits (43) | 63.56 | 150 | 236 |
|  | Yes (4) | 82.76 | 24 | 29 |
|  | NA (2) | 47.83 | 22 | 46 |
|  | **Total (50)** | **63.02** | **196** | **311** |
|  | **Feeding cat food** |  |  |  |
|  | Canned (4) | 68.18 | 30 | 44 |
|  | Canned or dry (4) | 50.00 | 6 | 12 |
|  | Dry (4) | 55.56 | 15 | 27 |
|  | No cat food (36) | 67.58 | 123 | 182 |
|  | NA (2) | 47.83 | 22 | 46 |
|  | **Total (50)** | **63.02** | **196** | **311** |
| **Food sources** |  |  |  |  |
|  | Labs (5) | 81.25 | 39 | 48 |
|  | Hunting (3) | 75.68 | 28 | 37 |
|  | Own breeding (19) | 66.67 | 84 | 126 |
|  | Slaughter (11) | 66.67 | 46 | 69 |
|  | Local Breeder (17) | 64.67 | 97 | 150 |
|  | Private owner (7) | 79.45 | 58 | 73 |
|  | Commercial animal food supplier (26) | 67.22 | 121 | 180 |
|  | NA (9) | 46.77 | 29 | 62 |
|  | **Total (50)** | **63.02** | **196** | **311** |
| **Food storage** |  |  |  |  |
|  | Cool (4) | 69.23 | 9 | 13 |
|  | Cool or Frozen (13) | 71.56 | 78 | 109 |
|  | Frozen (27) | 61.40 | 70 | 114 |
|  | NA (5) | 52.00 | 39 | 75 |
|  | **Total (50)** | **63.02** | **196** | **311** |
| **Length of freezing carcasses** |  |  |  |  |
|  | Sometimes < 1 week (14) | 76.19 | 64 | 84 |
|  | One week at least (9) | 48.00 | 12 | 25 |
|  | > 2 weeks (22) | 64.34 | 83 | 129 |
|  | NA (5) | 50.69 | 37 | 73 |
|  | **Total (50)** | **63.02** | **196** | **311** |
| **Separation of fruit and meat** |  |  |  |  |
|  | No separation (8) | 56.52 | 26 | 46 |
|  | Separation by processing (10) | 79.49 | 62 | 78 |
|  | Separation by different rooms (29) | 61.87 | 86 | 139 |
|  | NA (3) | 45.83 | 22 | 48 |
|  | **Total (50)** | **63.02** | **196** | **311** |
| **Water source** |  |  |  |  |
|  | Rain or Tap (3) | 75.00 | 6 | 8 |
|  | Tap (38) | 66.36 | 146 | 220 |
|  | Well or Tap (3) | 59.09 | 13 | 22 |
|  | Well (4) | 60.00 | 9 | 15 |
|  | NA (2) | 47.83 | 22 | 46 |
|  | **Total (50)** | **63.02** | **196** | **311** |
| **Clean water** |  |  |  |  |
|  | Daily (41) | 63.26 | 136 | 215 |
|  | Daily or Weekly (1) | 100.00 | 3 | 3 |
|  | Weekly (1) | 50.00 | 1 | 2 |
|  | Every 2nd week (4) | 79.07 | 34 | 43 |
|  | No change (1) | 00.00 | 0 | 2 |
|  | NA (2) | 47.83 | 22 | 46 |
|  | **Total (50)** | **63.02** | **196** | **311** |
| **Felids close by** |  |  |  |  |
|  | No Felids close by (16) | 55.32 | 26 | 47 |
|  | Yes (32) | 67.89 | 148 | 218 |
|  | NA (2) | 47.83 | 22 | 46 |
|  | **Total (50)** | **63.02** | **196** | **311** |
| **Marsupials close by** |  |  |  |  |
|  | No marsupials close by (31) | 65.00 | 117 | 180 |
|  | Yes (17) | 67.06 | 57 | 85 |
|  | NA (2) | 47.83 | 22 | 46 |
|  | **Total (50)** | **63.02** | **196** | **311** |
| **NWM close by** |  |  |  |  |
|  | No NWM close by (36) | 67.58 | 148 | 219 |
|  | Yes (12) | 56.52 | 26 | 46 |
|  | NA (3) | 47.83 | 22 | 46 |
|  | **Total (50)** | **63.02** | **196** | **311** |
| **Animal attendants care for more than one cat species** |  |  |  |  |
|  | No care for other felids (9) | 45.83 | 11 | 24 |
|  | Yes (39) | 67.64 | 163 | 241 |
|  | NA (2) | 47.83 | 22 | 46 |
|  | **Total (50)** | **63.02** | **196** | **311** |
| **Animal attendants care for cats plus NWM /Marsupials** |  |  |  |  |
|  | No care for cats + NWM/Marsupials (30) | 66.18 | 135 | 204 |
|  | Yes (18) | 63.93 | 39 | 61 |
|  | NA (2) | 47.83 | 22 | 46 |
|  | **Total (50)** | **63.02** | **196** | **311** |
| **Indoor housing** |  |  |  |  |
|  | No indoor housing (33) | 65.66 | 109 | 166 |
|  | Yes (15) | 65.66 | 65 | 99 |
|  | NA (2) | 47.83 | 22 | 46 |
|  | **Total (50)** | **63.02** | **196** | **311** |
| **Outdoor housing fenced in** |  |  |  |  |
|  | No fenced in outdoor housing (8) | 77.50 | 31 | 40 |
|  | Yes (40) | 63.56 | 143 | 225 |
|  | NA (2) | 47.83 | 22 | 46 |
|  | **Total (50)** | **63.02** | **196** | **311** |
| **Outdoor housing open top** |  |  |  |  |
|  | No open top outdoor housing (39) | 62.72 | 106 | 169 |
|  | Yes (9) | 70.83 | 68 | 96 |
|  | NA (2) | 47.83 | 22 | 46 |
|  | **Total (50)** | **63.02** | **196** | **311** |
| **Enclosure size < 20 m^2^** |  |  |  |  |
|  | Size not < 20 m^2^ (22) | 64.77 | 57 | 88 |
|  | Yes (26) | 66.10 | 117 | 177 |
|  | NA (2) | 47.83 | 22 | 46 |
|  | **Total (50)** | **63.02** | **196** | **311** |
| **Enclosure size 20-50 m^2^** |  |  |  |  |
|  | Size not between 20-50 m^2^ (14) | 68.89 | 31 | 45 |
|  | Yes (34) | 65.00 | 143 | 220 |
|  | NA (2) | 47.83 | 22 | 46 |
|  | **Total (50)** | **63.02** | **196** | **311** |
| **Enclosure size > 50 m^2^** |  |  |  |  |
|  | Size not > 50 m^2^ (32) | 66.87 | 109 | 163 |
|  | Yes (16) | 63.73 | 65 | 102 |
|  | NA (2) | 47.83 | 22 | 46 |
|  | **Total (50)** | **63.02** | **196** | **311** |
| **Mesh size < 2 cm** |  |  |  |  |
|  | Mesh size not < 2 cm (33) | 62.63 | 124 | 198 |
|  | Yes (15) | 74.63 | 50 | 67 |
|  | NA (2) | 47.83 | 22 | 46 |
|  | **Total (50)** | **63.02** | **196** | **311** |
| **Mesh size 2-5 cm** |  |  |  |  |
|  | Mesh size not between 2-5 cm (18) | 78.50 | 73 | 93 |
|  | Yes (30) | 58.72 | 101 | 172 |
|  | NA (2) | 47.83 | 22 | 46 |
|  | **Total (50)** | **63.02** | **196** | **311** |
| **Mesh size > 5 cm** |  |  |  |  |
|  | Mesh size not > 5cm (42) | 63.42 | 130 | 205 |
|  | Yes (6) | 73.33 | 44 | 60 |
|  | NA (2) | 47.83 | 22 | 46 |
|  | **Total (50)** | **63.02** | **196** | **311** |
| **No. of stray cats** |  |  |  |  |
|  | 0 (11) | 68.85 | 42 | 61 |
|  | 1 (9) | 61.29 | 19 | 31 |
|  | 2 (7) | 50.00 | 10 | 20 |
|  | 3 (4) | 66.67 | 6 | 9 |
|  | 4 (3) | 57.14 | 16 | 28 |
|  | 5 (5) | 68.42 | 26 | 38 |
|  | 6 (2) | 83.33 | 5 | 6 |
|  | 7 (1) | 81.82 | 27 | 33 |
|  | 8 (2) | 65.39 | 17 | 26 |
|  | 10 (1) | 100.00 | 2 | 2 |
|  | 15 (2) | 50.00 | 4 | 8 |
|  | NA (3) | 44.90 | 22 | 49 |
|  | **Total (50)** | **63.02** | **196** | **311** |
| **Number of institutions lived in** |  |  |  |  |
|  | > 1 institution (48) | 65.66 | 130 | 198 |
|  | Only 1 institution (22) | 58.56 | 65 | 111 |
|  | NA (2) | 50.00 | 1 | 2 |
|  | **Total (50)** | **63.02** | **196** | **311** |
| **Wood shavings** |  |  |  |  |
|  | No shavings (24) | 68.37 | 67 | 98 |
|  | Yes (24) | 64.07 | 107 | 167 |
|  | NA (2) | 47.83 | 22 | 46 |
|  | **Total (50)** | **63.02** | **196** | **311** |
| **Hay** |  |  |  |  |
|  | No Hay (33) | 63.43 | 111 | 175 |
|  | Yes (15) | 70.00 | 63 | 90 |
|  | NA (2) | 47.83 | 22 | 46 |
|  | **Total (50)** | **63.02** | **196** | **311** |
| **Straw** |  |  |  |  |
|  | No straw (23) | 65.71 | 69 | 105 |
|  | Yes (25) | 65.63 | 105 | 160 |
|  | NA (2) | 47.83 | 22 | 46 |
|  | **Total (50)** | **63.02** | **196** | **311** |
| **Sand** |  |  |  |  |
|  | No sand (27) | 71.76 | 94 | 131 |
|  | Yes (21) | 59.70 | 80 | 134 |
|  | NA (2) | 47.83 | 22 | 46 |
|  | **Total (50)** | **63.02** | **196** | **311** |
| **Other bedding materials** |  |  |  |  |
|  | No other materials (43) | 68.09 | 160 | 235 |
|  | Yes (5) | 56.67 | 14 | 30 |
|  | NA (2) | 47.83 | 22 | 46 |
|  | **Total (50)** | **63.02** | **196** | **311** |
| **Change of bedding material** |  |  |  |  |
|  | Daily (7) | 52.17 | 12 | 23 |
|  | Weekly (17) | 67.42 | 60 | 89 |
|  | Monthly (7) | 61.11 | 22 | 36 |
|  | Infrequently (16) | 67.83 | 78 | 115 |
|  | No change (1) | 100.00 | 2 | 2 |
|  | NA (2) | 47.83 | 22 | 46 |
|  | **Total (50)** | **63.02** | **196** | **311** |
| **Enrichment** |  |  |  |  |
|  | No enrichment (13) | 68.75 | 44 | 64 |
|  | Yes (35) | 64.68 | 130 | 201 |
|  | NA (2) | 47.83 | 22 | 46 |
|  | **Total (50)** | **63.02** | **196** | **311** |
| **Frequency of feces removal** |  |  |  |  |
|  | Daily (36) | 67.00 | 136 | 203 |
|  | Not Daily (12) | 61.29 | 38 | 62 |
|  | NA (2) | 47.83 | 22 | 46 |
|  | **Total (50)** | **63.02** | **196** | **311** |
| **Litterbox** |  |  |  |  |
|  | No litterbox (25) | 69.23 | 81 | 117 |
|  | Yes (23) | 62.84 | 93 | 148 |
|  | NA (2) | 47.83 | 22 | 46 |
|  | **Total (50)** | **63.02** | **196** | **311** |
| **Hygiene measures** |  |  |  |  |
|  | No hygiene measures (11) | 62.79 | 54 | 86 |
|  | Yes (37) | 67.04 | 120 | 179 |
|  | NA (2) | 47.83 | 22 | 46 |
|  | **Total (50)** | **63.02** | **196** | **311** |
| **Hand wash facilities** |  |  |  |  |
|  | No hand wash facilities (35) | 66.12 | 121 | 183 |
|  | Yes (13) | 64.63 | 53 | 82 |
|  | NA (2) | 47.83 | 22 | 46 |
|  | **Total (50)** | **63.02** | **196** | **311** |
| **Using gloves** |  |  |  |  |
|  | No gloves (28) | 72.38 | 131 | 181 |
|  | Yes (20) | 51.19 | 43 | 84 |
|  | NA (2) | 47.83 | 22 | 46 |
|  | **Total (50)** | **63.02** | **196** | **311** |
| **Disinfectant footbath** |  |  |  |  |
|  | No footbath (30) | 65.03 | 106 | 163 |
|  | Yes (18) | 66.67 | 68 | 102 |
|  | NA (2) | 47.83 | 22 | 46 |
|  | **Total (50)** | **63.02** | **196** | **311** |
| **Shoe cover** |  |  |  |  |
|  | No shoe covers (47) | 66.54 | 173 | 260 |
|  | Yes (1) | 20.00 | 1 | 5 |
|  | NA (2) | 47.83 | 22 | 46 |
|  | **Total (50)** | **63.02** | **196** | **311** |
| **Pest control** |  |  |  |  |
|  | No pest control (4) | 72.22 | 39 | 54 |
|  | Yes (43) | 64.90 | 135 | 208 |
|  | NA (3) | 44.90 | 22 | 49 |
|  | **Total (50)** | **63.02** | **196** | **311** |
| **Pest control frequency in month** |  |  |  |  |
|  | 0 (3) | 53.85 | 7 | 13 |
|  | 0.25 (1) | 100.00 | 4 | 4 |
|  | 1 (21) | 58.67 | 44 | 75 |
|  | 2 (3) | 71.43 | 15 | 21 |
|  | 3 (5) | 68.75 | 11 | 16 |
|  | 6 (3) | 80.00 | 4 | 5 |
|  | 12 (1) | 66.67 | 2 | 3 |
|  | Infreq (3) | 78.38 | 29 | 37 |
|  | NA (10) | 58.39 | 80 | 137 |
|  | **Total (50)** | **63.02** | **196** | **311** |
| **Traps for Pest control** |  |  |  |  |
|  | No traps used (19) | 69.01 | 98 | 142 |
|  | Yes (28) | 63.33 | 76 | 120 |
|  | NA (3) | 44.90 | 22 | 49 |
|  | **Total (50)** | **63.02** | **196** | **311** |
| **Poison for Pest control** |  |  |  |  |
|  | No poison used (37) | 67.12 | 49 | 73 |
|  | Yes (10) | 66.14 | 125 | 189 |
|  | NA (3) | 44.90 | 22 | 49 |
|  | **Total (50)** | **63.02** | **196** | **311** |
| **Cats used for Pest control** |  |  |  |  |
|  | No cats used (33) | 66.32 | 128 | 193 |
|  | Yes (14) | 66.67 | 46 | 69 |
|  | NA (3) | 44.90 | 22 | 49 |
|  | **Total (50)** | **63.02** | **196** | **311** |
| **Vaccination** |  |  |  |  |
|  | No vaccination (7) | 46.67 | 14 | 30 |
|  | Yes (37) | 64.58 | 124 | 192 |
|  | NA (6) | 65.17 | 58 | 89 |
|  | **Total (50)** | **63.02** | **196** | **311** |
| **Cat flu Vaccination** |  |  |  |  |
|  | No cat flu vaccination (12) | 52.17 | 24 | 46 |
|  | Yes (32) | 64.77 | 114 | 176 |
|  | NA (6) | 65.17 | 58 | 89 |
|  | **Total (50)** | **63.02** | **196** | **311** |
| **Parvovirus Vaccination** |  |  |  |  |
|  | No parvovirus vaccination (13) | 58.93 | 33 | 56 |
|  | Yes (31) | 63.25 | 105 | 166 |
|  | NA (6) | 65.17 | 58 | 89 |
|  | **Total (50)** | **63.02** | **196** | **311** |
| **Rabies Vaccination** |  |  |  |  |
|  | No rabies vaccination (27) | 53.19 | 75 | 141 |
|  | Yes (17) | 77.78 | 63 | 81 |
|  | NA (6) | 65.17 | 58 | 89 |
|  | **Total (50)** | **63.02** | **196** | **311** |
| **Leucosis Vaccination** |  |  |  |  |
|  | No leucosis vaccination (29) | 60.87 | 84 | 138 |
|  | Yes (15) | 64.29 | 54 | 84 |
|  | NA (6) | 65.17 | 58 | 89 |
|  | **Total (50)** | **63.02** | **196** | **311** |
| **FIP Vaccination** |  |  |  |  |
|  | No FIP vaccination (31) | 61.77 | 105 | 170 |
|  | Yes (13) | 63.46 | 33 | 52 |
|  | NA (6) | 65.17 | 58 | 89 |
|  | **Total (50)** | **63.02** | **196** | **311** |
| **Check for endoparasites** |  |  |  |  |
|  | No check for endoparasites (6) | 63.64 | 35 | 55 |
|  | Yes (39) | 62.13 | 105 | 169 |
|  | NA (5) | 64.37 | 56 | 87 |
|  | **Total (50)** | **63.02** | **196** | **311** |
| **Frequency of endoparasite checks in month** |  |  |  |  |
|  | 0 (1) | 00.00 | 0 | 1 |
|  | 1 (1) | 00.00 | 0 | 3 |
|  | 2 (3) | 71.43 | 10 | 14 |
|  | 3 (12) | 58.54 | 24 | 41 |
|  | 6 (13) | 60.00 | 18 | 30 |
|  | 12 (7) | 61.11 | 22 | 36 |
|  | NA (13) | 65.59 | 122 | 186 |
|  | **Total (50)** | **63.02** | **196** | **311** |
| **Deworming interval in month** |  |  |  |  |
|  | 3 (7) | 63.16 | 36 | 57 |
|  | 4 (1) | 62.50 | 5 | 8 |
|  | 6 (21) | 60.00 | 45 | 75 |
|  | 12 (3) | 100.00 | 20 | 20 |
|  | NA (18) | 59.60 | 90 | 151 |
|  | **Total (50)** | **63.02** | **196** | **311** |
| **No. of death** |  |  |  |  |
|  | 0 (21) | 65.12 | 56 | 86 |
|  | 1 (11) | 54.17 | 13 | 24 |
|  | 2 (1) | 100.00 | 2 | 2 |
|  | 3 (2) | 66.67 | 22 | 33 |
|  | 4 (2) | 80.95 | 34 | 42 |
|  | 5 (5) | 73.68 | 14 | 19 |
|  | 7 (1) | 40.00 | 4 | 10 |
|  | 9 (1) | 50.00 | 2 | 4 |
|  | 10 (1) | 80.00 | 4 | 5 |
|  | 12 (1) | 62.50 | 10 | 16 |
|  | 16 (1) | 50.00 | 11 | 22 |
|  | NA (3) | 50.00 | 24 | 48 |
|  | **Total (50)** | **63.02** | **196** | **311** |
| **Age Categories** |  |  |  |  |
|  | <5 Years (33) | 48.94 | 69 | 141 |
|  | >5 Years (44) | 78.34 | 123 | 157 |
|  | NA (6) | 30.77 | 4 | 13 |
|  | **Total (50)** | **63.02** | **196** | **311** |
| **Rearing** |  |  |  |  |
|  | Parent (48) | 62.41 | 176 | 282 |
|  | Hand (11) | 69.23 | 9 | 13 |
|  | Foster (1) | 100.00 | 1 | 1 |
|  | NA (11) | 66.67 | 10 | 15 |
|  | **Total (50)** | **63.02** | **196** | **311** |
|  | **Sex** |  |  |  |
|  | Male (45) | 67.09 | 106 | 158 |
|  | Female (46) | 59.60 | 90 | 151 |
|  | NA (2) | 00.00 | 0 | 2 |
|  | **Total (50)** | **63.02** | **196** | **311** |
| **Species** |  |  |  |  |
|  | Asian golden cat (2) | 50.00 | 1 | 2 |
|  | Black-footed cat (3) | 26.67 | 4 | 15 |
|  | Fishing cat (15) | 55.00 | 22 | 40 |
|  | Geoffroy’s cat (14) | 48.49 | 16 | 33 |
|  | Jaguarondi (5) | 55.56 | 5 | 9 |
|  | Margay (7) | 47.37 | 9 | 19 |
|  | Oncilla (3) | 66.67 | 6 | 9 |
|  | Pallas’ cat (22) | 90.39 | 47 | 52 |
|  | Rusty-spotted cat (3) | 96.43 | 27 | 28 |
|  | Sand cat (15) | 54.02 | 47 | 87 |
|  | Others (5) | 70.59 | 12 | 17 |
|  | **Total (50)** | **63.02** | **196** | **311** |
| **No. of felids** |  |  |  |  |
|  | 1-16 (24) | 63.42 | 163 | 257 |
|  | ≥17 (26) | 61.11 | 33 | 54 |
|  | **Total (50)** | **63.02** | **196** | **311** |
|  | **No. of litters within 5 Years** |  |  |  |
|  | 0 (12) | 52.78 | 19 | 36 |
|  | 1-9 (20) | 71.58 | 68 | 95 |
|  | ≥10 (18) | 60.56 | 109 | 180 |
|  | **Total (50)** | **63.02** | **196** | **311** |
| **No. of litters within 1 Year** |  |  |  |  |
|  | 0 (17) | 50.00 | 25 | 50 |
|  | 1-2 (17) | 69.74 | 53 | 76 |
|  | ≥3 (16) | 63.78 | 118 | 185 |
|  | **Total (50)** | **63.02** | **196** | **311** |
| **No. of litters within 5 Years** |  |  |  |  |
|  | 0 (12) | 52.78 | 19 | 36 |
|  | 1-9 (20) | 71.58 | 68 | 95 |
|  | ≥10 (18) | 60.56 | 109 | 180 |
|  | **Total (50)** | **63.02** | **196** | **311** |
