# Supplementary material for: Toxoplasma gondii in small exotic felids from zoos in Europe and the Middle East: serological prevalence and risk factors
Source: Parasit Vectors. 2019 Sep 11;12:449. doi: 10.1186/s13071-019-3706-2 (PMC6737647; doi:10.1186/s13071-019-3706-2)
Supplement: Supplementary file 7 — Additional file 7: Table S6. Results of factor analysis 1. Table S7. Results of factor analysis 2. Table S8. Results of factor analysis 3. [file 13071_2019_3706_MOESM7_ESM.docx]

**Additional file 7: Table S6** Details of factor analysis 1. Absolute factor loadings > 0.4 in bold.

|  | **Factor1** | **Factor2** | **Factor3** | **Factor4** | **Factor5** | **Factor6** | **Factor7** | **Factor8** |
| --- | --- | --- | --- | --- | --- | --- | --- | --- |
| Age |  | 0.191 |  |  |  |  |  |  |
| Sex |  |  |  |  |  |  | -0.161 |  |
| Feeding-Mice | **0.923** |  |  | -0.136 |  | 0.282 | -0.165 | 0.106 |
| Feeding-Cattle |  | **0.808** | 0.223 |  |  |  | **0.523** |  |
| Feeding-Fowl | **0.609** | 0.181 | -0.207 |  |  |  | 0.251 | 0.258 |
| NWMCloseby | 0.205 |  |  |  |  | **0.666** | 0.173 | 0.209 |
| Wearing gloves |  |  |  |  |  | 0.107 |  | **0.414** |
| Outdoor_Fencedin-allsides | 0.265 |  | -0.286 |  | -0.13 | 0.156 | 0.282 |  |
| MeshSize |  | -0.15 | -0.264 | **-0.524** | -0.159 | -0.132 |  | 0.244 |
| Rabies vaccination | -0.337 | -0.281 |  | **0.888** |  | -0.1 |  |  |
| DewormingInterval-Months | -0.175 |  |  |  | **0.95**7 |  | 0.154 | -0.106 |
| Feeding-Ruminants |  | **0.961** |  | 0.106 |  |  | 0.198 |  |
| Feeding-Rodents | **0.971** |  |  | -0.146 | -0.152 |  |  |  |
| LittersWithin1Year | -0.193 | 0.244 | **0.768** |  |  | 0.162 |  | -0.326 |
| LittersWithin5Years |  | -0.105 | **0.950** |  |  | -0.202 | -0.111 | 0.129 |

**Additional file 7: Table S7** Details of factor analysis 2. Absolute factor loadings > 0.4 in bold.

|  | **Factor1** | **Factor2** | **Factor3** | **Factor4** | **Factor5** | **Factor6** |
| --- | --- | --- | --- | --- | --- | --- |
| Age |  |  | **0.996** |  |  |  |
| Sex |  |  |  |  |  | -0.287 |
| Feeding-Mice | -0.152 | 0.329 |  |  | -0.233 |  |
| Feeding-Cattle | **0.501** |  | 0.103 |  |  | 0.204 |
| NWMCloseby |  | **0.992** |  |  |  |  |
| Wearing gloves |  | 0.21 |  | 0.12 |  | 0.19 |
| Outdoor_Fencedin-allsides | -0.102 | 0.203 | -0.11 |  | -0.113 | **0.582** |
| MeshSize | -0.183 |  |  | **0.974** | -0.106 |  |
| DewormingInterval-Months |  | -0.143 |  |  | **0.930** |  |
| LittersWithin1Year | **0.927** | -0.114 |  | -0.189 |  | -0.254 |

**Additional file 7: Table S8** Details of factor analysis 3. Absolute factor loadings > 0.4 in bold.

|  | **Factor1** | **Factor2** | **Factor3** | **Factor4** | **Factor5** | **Factor6** |
| --- | --- | --- | --- | --- | --- | --- |
| Age |  | **0.994** |  |  |  |  |
| Sex |  |  |  |  | -0.361 | -0.287 |
| Feeding-Mice | 0.216 |  | **0.485** |  |  |  |
| Feeding-Cattle |  | 0.115 | -0.142 | -0.181 | 0.267 | 0.204 |
| NWMCloseby | **0.966** |  | 0.241 |  |  |  |
| Using gloves | 0.179 |  |  | 0.363 | 0.132 | 0.19 |
| Outdoor_Fencedin-allsides | 0.161 | -0.141 | 0.214 | 0.209 | **0.444** | **0.582** |
| MeshSize | -0.129 |  | 0.204 | **0.460** | -0.172 |  |
| DewormingInterval-Months |  |  | **-0.515** | -0.196 |  |  |
